# Comprehensive analysis of a new prognosis signature based on histone deacetylases in clear cell renal cell carcinoma

**DOI:** 10.1002/cam4.4156

**Published:** 2021-07-26

**Authors:** Fajuan Cheng, Bin Zheng, Jianwei Wang, Guiting Zhao, Zhongshun Yao, Zhihong Niu, Wei He

**Affiliations:** ^1^ Department of Nephrology Shandong Provincial Hospital Affiliated to Shandong University Jinan Shandong China; ^2^ Department of Nephrology Shandong Provincial Hospital Cheeloo College of Medicine Shandong University Jinan Shandong China; ^3^ Cheeloo College of Medicine Shandong University Jinan Shandong P.R. China; ^4^ Department of Urology Shandong Provincial Hospital Affiliated to Shandong First Medical University Jinan Shandong P.R. China; ^5^ Department of Urology Shandong Provincial Hospital Affiliated to Shandong University Jinan Shandong P.R. China; ^6^ Department of Urology Shandong Provincial ENT Hospital Affiliated to Shandong University Jinan Shandong P.R. China

**Keywords:** histone deacetylase, overall survival, prognosis, renal cell carcinoma, signature

## Abstract

Histone deacetylases (HDAC) family is vital for tumorigenesis and tumor progression. However, the exact role of the HDAC family in clear cell renal cell carcinoma (ccRCC) remains unclear. Based on The Cancer Genome Atlas (TCGA), International Cancer Genome Consortium (ICGC), and The Human Protein Atlas (HPA) database, we investigated and validated the expression profile, clinical significance and prognostic value of HDAC family members in ccRCC. Moreover, we further explored the correlation between HDACs and tumor microenvironment, tumor stemness, drug activity and immune subtype. The *HDAC8*, *HDAC10,* and *HDAC11* manifested potential clinical value for prognosis, and the correlation analyses reveals underlying molecular mechanisms, which deserve further investigation for ccRCC. This Integrated bioinformatics analysis, based on transcriptomics and proteomics, implied that *HDAC8*, *HDAC10,* and *HDAC11* may serve as potential molecular biomarkers and therapeutic targets for ccRCC, but some underlying molecular mechanisms still need to be elucidated.

## Graphical abstract

Risk model consisting of HDAC8, HDAC10, and HDAC11 have a favorable role of risk prediction in ccRCC. Immune‐associated functions and pathways were enriched in HDACs, and most of HDACs are related to tumor stemness, stromal cell, and immune cells. Several clinical characteristics are associated with HDACs expression and the activity of some commonly used drugs (such as oxaliplatin, vorinostat, temsirolimus) are also influenced by HDACs.

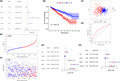

## 1 INTRODUCTION

Kidney cancer is a common urinary carcinoma, with appropriately 73,820 patients newly diagnosed and 14,770 patients die of it in the United States.^1^ As the most common histological subtype of renal cell carcinoma (RCC), clear cell carcinoma (ccRCC) always leads to a poor survival rate. It is estimated that nearly 25%–30% ccRCC patients have metastasized by the initial diagnosis time.^2^, ^3^

With the understanding of human genetics, medical oncology had revolutionized. Epigenetics, a heritable alteration in gene expression, make genetic material package effectively.^4^ As one of three interlinked epigenetic modifications, histone covalent modification, especially histone acetylation, plays an indispensable role in the expression status of promoters.^5^ Recently, numbers of epigenetic mechanism of oncogenesis have been analyzed to its finer detail. In RCC, histone modification is strongly associated with the increased risk of poor prognosis. For example, reduction in H3Ac, H4Ac, H3K18Ac, and H3K27me are related to poor clinical outcomes in RCC patients, such as recurrence, metastasis, worse cancer‐specific survival, and progression‐free survival.^6^, ^7^, ^8^

Histone deacetylases (HDACs), consisting of 18 conserved genes, are divided into four classes: class Ⅰ (*HDAC1*, *HDAC2*, *HDAC3*, *HDAC8*), class Ⅱ (*HDAC4*, *HDAC5*, *HDAC6*, *HDAC7*, *HDAC9*, *HDAC10*), class Ⅲ (*SIRT1* – *SIRT7*), and class Ⅳ (*HDAC11*).^9^ Previous evidence suggested that *HDACs* in class Ⅰ overexpressed in ccRCC and *HDACs* in class Ⅱ regulated ccRCC biological functions.^10^, ^11^ However, research concerning about *HDACs* in ccRCC biology and prognosis is still lacking. Because *HDAC* inhibitors mainly target class Ⅰ, Ⅱ, and Ⅳ, which are also known as classical *HDACs*,^12^ we evaluated these 11 genes in this study. We hope that this study could contribute to the understanding of novel molecular therapeutic targets for ccRCC patients.

## 2 MATERIALS AND METHODS

### 2.1 Data collection and analysis

The expression data and clinical information of ccRCC and 33 cancers were downloaded directly from the ICGC (https://dcc.icgc.org/) and UCSC Xena database (https://xena.ucsc.edu/). Stemness score data and immune subtype were also downloaded from the UCSC Xena database.^40^ All gene expression data were normalized by “limma” package.

We assessed the differentially expressed genes (DEGs) in paired tumor as well as non‐tumor tissues by the “limma” R package and visualized by “pheatmap” and “vioplot” R package. Correlation between *HDACs* and stemness was performed by “corrplot” package. Correlation between *HDACs* and immune subtype was visualized by “ggplot2” package. The protein–protein interaction (PPI) network was performed for all *HDACs* via the STRING database (http://string‐db.org/).

LASSO regression analysis, univariate, and multivariate Cox regression analyses were separately conducted by "glmnet" and “survival” package.^41^ Gene Ontology (GO) and Kyoto Encyclopedia of Genes and Genomes (KEGG) analyses were performed through "clusterProfiler" package. Single‐sample gene set enrichment analysis (ssGSEA) was performed by the "gsva" package.

### 2.2 Construction and validation of the prognostic signature

The prognostic‐related genes were selected after performing univariate Cox analysis with *p* < 0.05. To minimize the risk of overfitting and choose optimal genes, LASSO regression analysis was conducted. Then, the median value of the risk score was calculated by this formula: Risk score = $\sum_{1}^{n}Coef_{n}\times x_{n}$ (Coef_n_ is the coefficient and x_n_ is the expression level of each genes). Based on median risk scores, ccRCC samples were stratified into high‐ and low‐risk sets. Afterward, the Kaplan–Meier (K‐M) curve, PCA analysis, and distribution of risk scores were executed to assess the accuracy of the established prognostic model. We verified our model via the HPA database (https://www.proteinatlas.org/).

### 2.3 GSCAlite database, CellMiner database, Oncomine database, and UALCAN analysis

We used GSCALite database (http://bioinfo.life.hust.edu.cn/web/GSCALite/) to explore SNV and CNV of *HDACs* in pan‐cancer and the degree of Cancer pathway activity.^42^ The UALCAN online tool (http://ualcan.path.uab.edu/) was utilized to analyze protein expression in ccRCC.^43^ CellMiner database (https://discover.nci.nih.gov/cellminer/home.do) was utilized to integrate *HDACs* and pharmacological data.^44^ Oncomine dataset (www.oncomine.org) was employed to compare the expression profile of *HDACs* in pan‐cancers.^45^

### 2.4 ESTIMATE algorithm

We used ESTIMATE (Estimation of Stromal and Immune cells in Malignant Tumor tissues using Expression data) algorithm to measure stromal scores and immune scores to predict the infiltration of stromal and immune cells among 33 tumors (https://portal.gdc.cancer.gov/).^45^

### 2.5 Statistical analysis

Mann–Whitney test was utilized to measure gene expression level. We eliminate samples that clinical information is lost or unknown. The K–M curve with log‐rank test was adopted in survival analysis. Statistical analysis was performed with R packages (R version 4.0.1). A two‐tailed *p* < 0.05 was considered significant.

### 2.6 Nomenclature

aDC: Activated dendritic cell; APC: Antigen‐presenting cell; CCR: Cytokine–cytokine receptor; CI: Confidence interval; EMT: Epithelial‐Mesenchymal Transition; FDR: False discovery rate; HLA: Human leukocyte antigen; HR: Hazard ratio; iDC: Immature dendritic cell; LASSO: Least absolute shrinkage and selection operator; MHC: Major histocompatibility complex; PCA: Principal component analysis; Tfh: T follicular helper cell; TIL: Tumor‐Infiltrating Lymphocyte.

## 3 RESULTS

### 3.1 The differential expression pattern of HDACs in pan‐cancers

**FIGURE 1:**
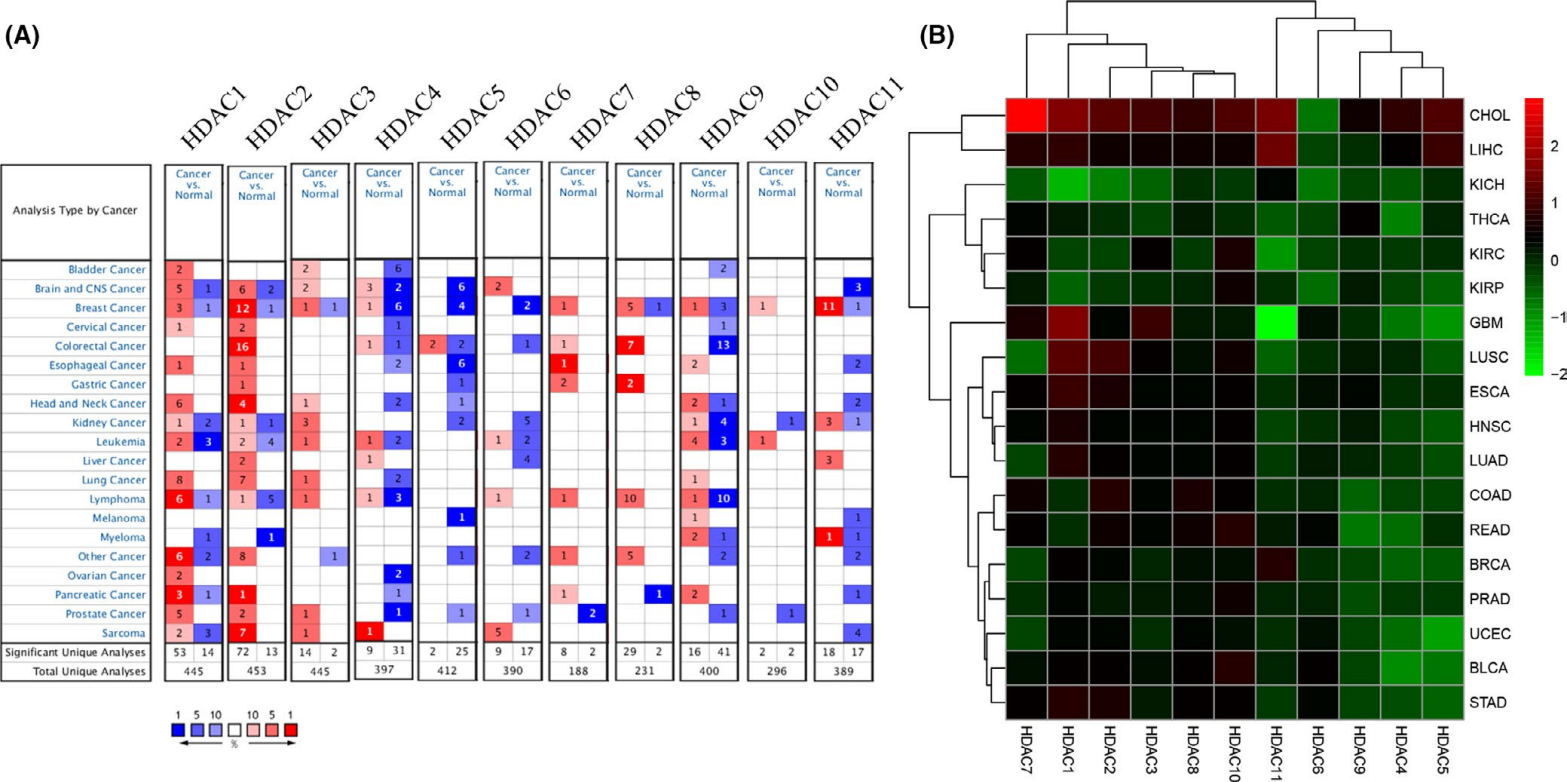
Pan‐cancer analysis of HDACs expression by the Oncomine database (A) and the TCGA database (B). Red grids (*p* < 0.05, FDR >1.5) and boxplots in cancer tissue; Blue grids in normal tissues. *p < 0.05; **p < 0.01; ***p < 0.001

We investigated the mRNA expression by using Oncomine database. As can be seen from Figure 1A, several genes (*HDAC1*, *HDAC2*, *HDAC3*, *HDAC7*, *HDAC8*, *HDAC9*) express highly in most cancers, such as brain and CNS cancer, kidney cancer, breast cancer, and leukemia, which presents its potential role in pan‐cancer. Besides, we also analyzed the expression level of *HDACs* proteins by UALCAN database in breast cancer, colon cancer, ovarian cancer, clear call renal cancer, uterine corpus endometrial carcinoma, and lung adenocarcinoma. The results of boxplot also show that there is a significantly different expression level in *HDAC* family, which confirmed the above results (Figure [Link], [Link], [Link], [Link], [Link], [Link]). We also verified these results by downloading mRNA expression data from TCGA database. As shown in Figures 1B and S7, these results are consistent with results from the Oncomine database.

### 3.2 Genetic alterations of HDACs in pan‐cancers

Due to gene mutation is instrumental in tumorigenesis, we illustrated the single nucleotide variations (SNV) and copy number variations (CNV) of *HDACs* in pan‐cancers by utilizing the GSCAlite database. First, as shown in Figure S8A, *HDAC9* and *HDAC7* have heterozygous amplifications in most of cancers, and *HDAC8*, *HDAC6*, *HDAC4*, *HDAC1*, *HDAC2*, *HDAC10,* and *HDAC11* have apparently heterozygous mutations in the majority of cancers as well. The results from SNV analysis indicate that *HDAC9*, *HDAC4*, *HDAC6*, *HDAC5*, *HDAC7*, *HDAC3*, *HDAC2*, *HDAC11*, *HDAC10*, and *HDAC8* are the top 10 mutated genes, with mutation rates from 7% to 32% (Figure S8B,C). Besides, the missense mutation occupies the most part in numerous types of mutations and appears more frequently in uterine endometrial carcinoma (Figure S8B,C).

### 3.3 Prognostic significance of HDACs in pan‐cancers

Considering the above results of *HDACs* in pan‐cancers and limited prognostic data of *HDAC* family, we then assessed the prognostic value of *HDACs* in pan‐cancers through utilizing overall survival (OS) data from the TCGA. This revealed that in most of cancers, low expression of *HDACs* could lead to a better survival condition (Figure S9). However, in certain cancers, such as adrenocortical carcinoma, bladder urothelial, cervical squamous cell carcinoma, and cholangiocarcinoma, several HDAC family members act as favor prognosis biomarkers. For example, in RCC (including chromophobe carcinoma, ccRCC, and papillary cell carcinoma), low expression of *HDAC1*, *HDAC2*, *HDAC3*, *HDAC8*, *HDAC10* and high expression of *HDAC5*, *HDAC7*, *HDAC11* have longer survival time.

The existing evidence suggested that various *HDAC* inhibitors, including LBH589 and OBP‐801, could promote RCC cell apoptosis and ameliorate the outcomes of RCC patients.^13^, ^14^ Given the potential clinical value of the HDAC family in ccRCC, we next conducted a comprehensive analysis of HDACs in ccRCC.

### 3.4 HDAC family expression in ccRCC

**FIGURE 2:**
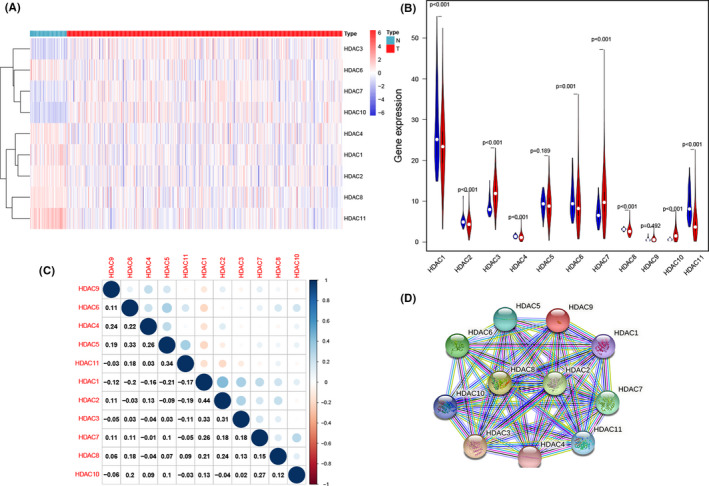
The expression and correlation of HDAC family in ccRCC. (A, B) The heatmap and violin plot of the expression data from TCGA. (C) The correlation network of HDACs. (D) The PPI network from the STRING database. *p < 0.05; **p < 0.01; ***p < 0.001

The expression heatmap and violin plot indicate the mRNA expression level of *HDAC* family (Figure 2A,B). Most of genes (9/11, 81.8%) show significantly difference expression in ccRCC samples, and among these genes, *HDAC3*, *HDAC7,* and *HDAC10* have higher expression level, compared with normal samples. Moreover, the expression of *HDAC1*, *HDAC2*, *HDAC4*, *HDAC6*, *HDAC8,* and *HDAC11* present a significant decrease in ccRCC samples. For the last two genes, *HDAC5* and *HDAC9*, the apparently statistic difference was not observed between normal samples and ccRCC samples. The above results suggest that HDAC family may have a huge influence on ccRCC.

We also analyzed the interactive relationship among all *HDACs* genes. The results suggested that most of these genes had a positive correlation and the most significant paired genes are *HDAC2* and *HDAC1* (*r* = 0.44) (Figure 2C). The PPI network shows that *HDAC2* and *HDAC8* are hub genes (Figure 2D).

### 3.5 Construction of the HDAC‐based risk signature

**FIGURE 3:**
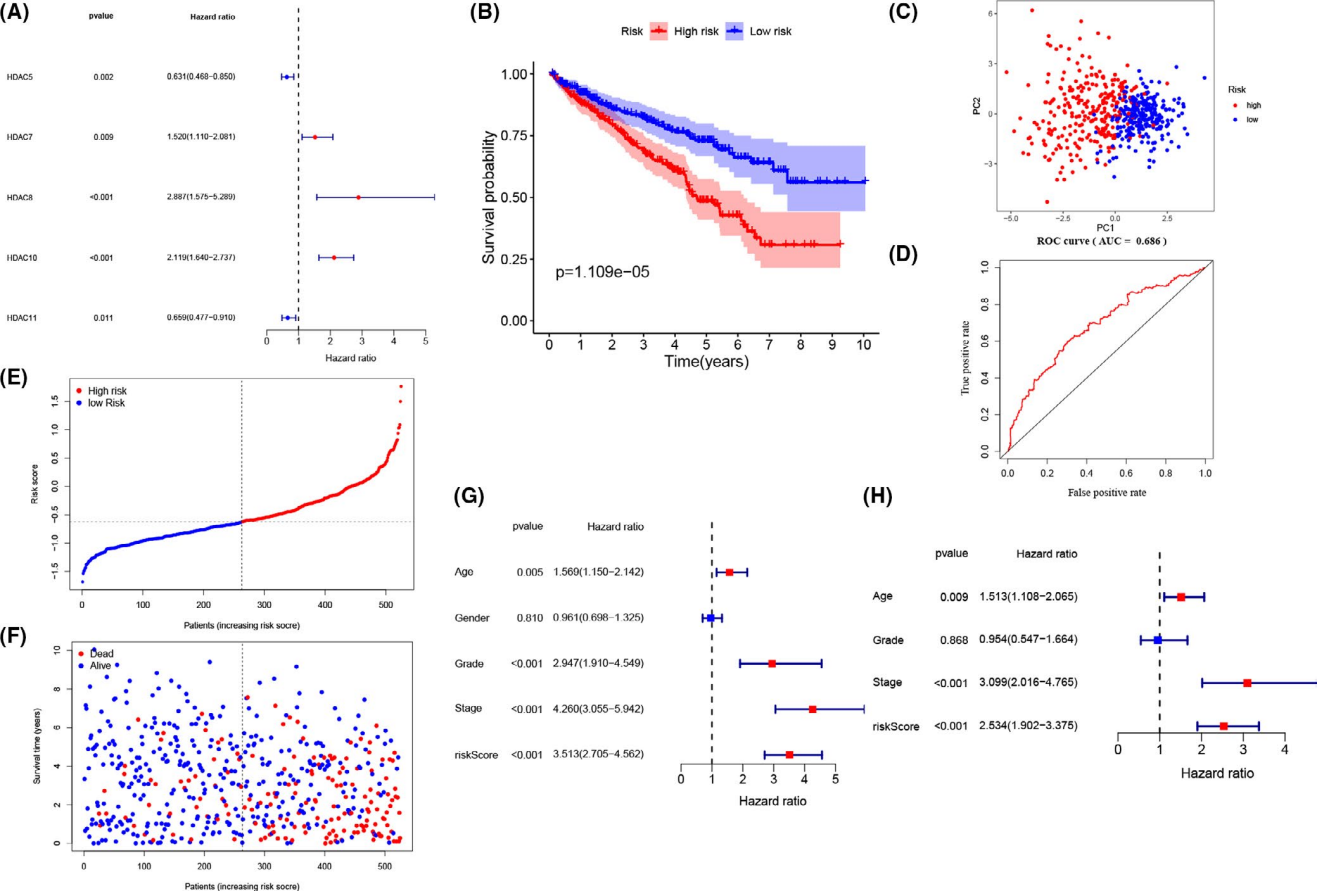
Construction of the HDAC‐based risk signature in TCGA cohort. (A) Construction of univariate Cox analysis. (B) the K–M analysis for the OS of patients in the high‐ and low‐risk group. C. PCA plot of the TCGA cohort. (D) The ROC curve in TCGA set. (E, F) The distribution of the risk scores and corresponding overall survival status. (G, H) The univariate and multivariate Cox analyses regarding OS

Due to limited study investigated the potential prognostic value of *HDACs* in ccRCC. we applied the univariate Cox regression analysis with *p* < 0.05 to select the prognostic‐related genes, and the results reveal that the expression of *HDAC7*, *HDAC8,* and *HDAC10* are positively correlated with survival rates and the expression of *HDAC5*, *HDAC11* are negatively correlated with survival rates in ccRCC patients (Figure 3A). After putting all these genes into LASSO regression analysis, the final signature was built by *HDAC8*, *HDAC10* and *HDAC11*. All patients were divided into low and high sets based on median risk scores. Next, we employed K–M analysis with a 95% confidence interval. The results clearly indicate that patients in low‐risk set have a better survival time (*p* < 0.05) (Figure 3B). Then, the ROC curve demonstrates that the risk signature has an acceptable efficiency (AUC = 0.686) and the PCA analysis based on the risk signature successfully distinguish two risk set (Figure 3C,D). The risk scores of three genes and corresponding expression profiles are shown in Figure 3E,F. Overall, the results demonstrate that the three‐gene risk signature could effectively filter out high‐risk ccRCC patients with poor clinical outcomes. From Figure 3G,H, we can observe that patients’ age (HR = 1.513, 95% CI [1.108–2.065], *p* = 0.009), tumor stage (HR = 3.099, 95% CI [2.016–4.765], *p* < 0.001), and risk score (HR = 2.534, 95% CI [1.902–3.375], *p* < 0.001) are associated with worse OS.

### 3.6 Validation of the HDAC‐based risk signature

**FIGURE 4:**
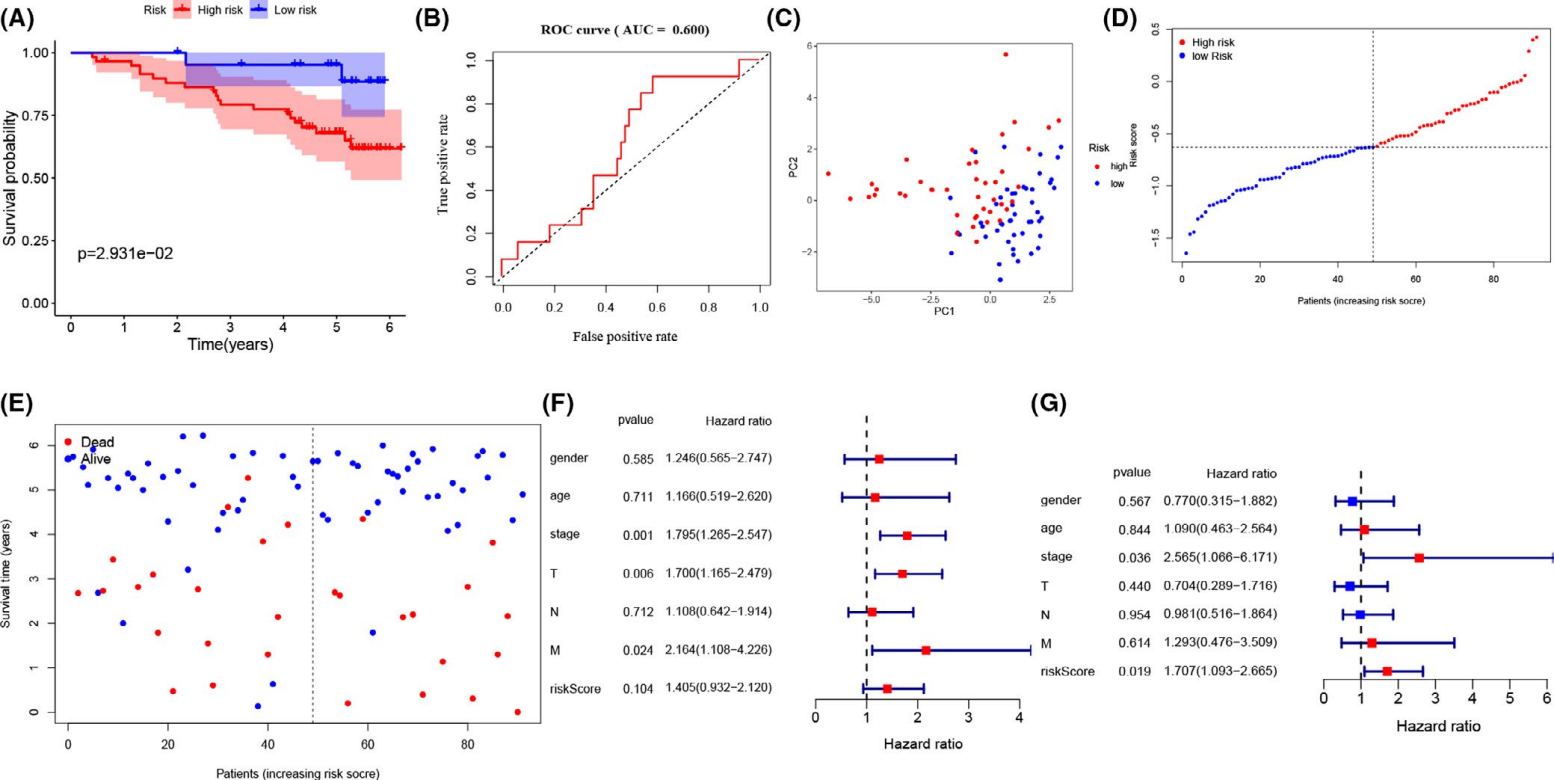
Validation of the HDAC‐based risk signature in ICGC cohort. (A) the K–M analysis for the OS of patients in the high‐ and low‐risk group. (B) The ROC curve in ICGC set. (C) PCA plot of the ICGC cohort. (D, E) The distribution of the risk scores and its corresponding overall survival status. (F, G) The univariate and multivariate Cox analyses regarding OS

To valid the above results, we used 92 ccRCC patients with complete clinical information from ICGC database, which contains. We observed that the risk model demonstrated the same trend when ccRCC patients from ICGC database were separated into high‐ and low set at median risk scores (Figure 4A–E). The results from K–M analysis and the risk scores present that high‐risk patients have worse survival condition and the ROC curve presents that risk score has an acceptable predictive ability (AUC = 0.600) (Figure 4A,B). Moreover, we found that in the ICGC validation set, tumor stage (HR = 2.565, 95% CI [1.066–6.171], *p* = 0.036) and risk score (HR = 1.707, 95% CI [1.093–2.665], *p* = 0.019) are correlated with worse OS (Figure 4F,G). Finally, we validated the immunohistochemistry pattern by utilizing the HPA database.

**FIGURE 5:**
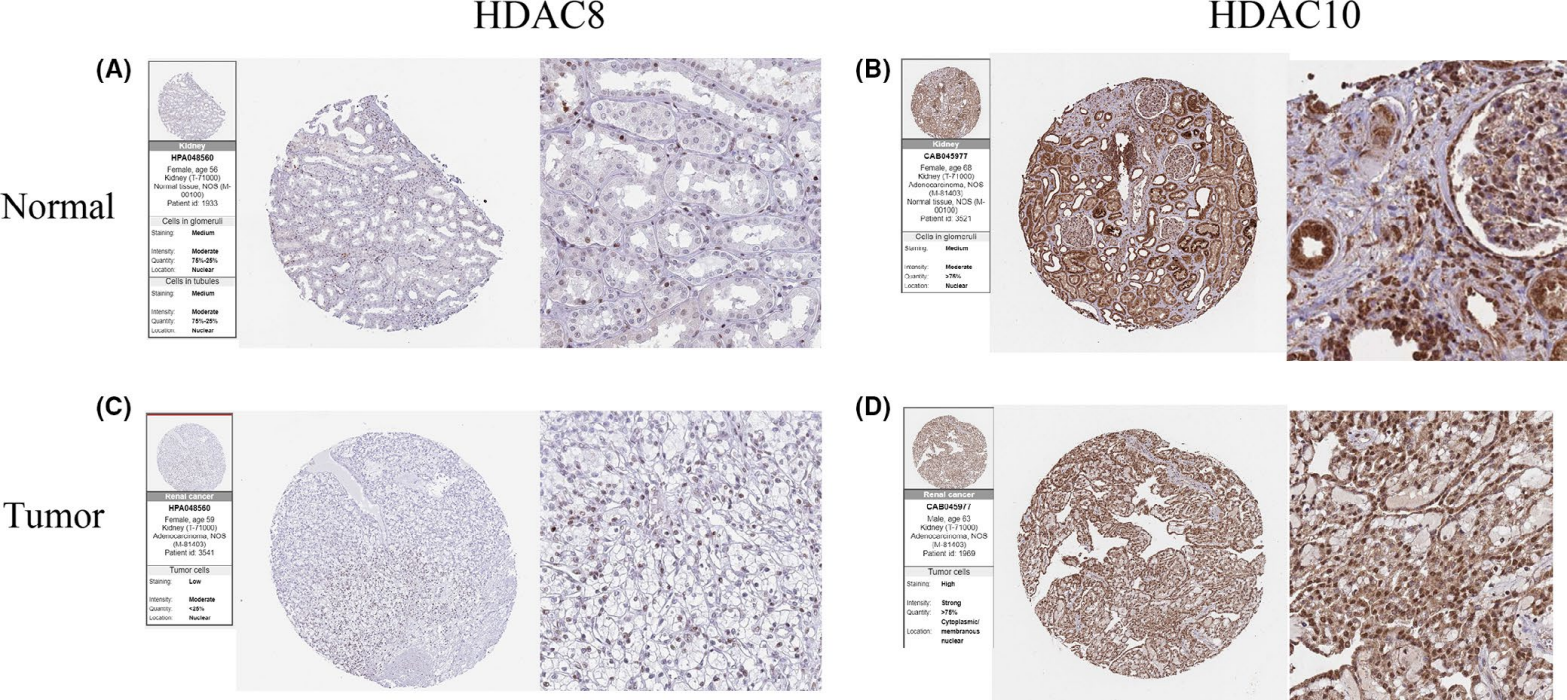
The IHC expression pattern of HDAC8 and HDAC10 in RCC tissues and normal tissues. (A, B) IHC of Normal kidney tissue. (C, D) IHC of RCC tissue

We noticed that normal kidney tissue staining of *HDAC8* exhibits medium staining in nuclear of tubules cells (Figure 5A). Instead, the weak staining was located in the nuclear of tumor cells. (Figure 5C). For *HDAC10*, moderate staining patterns were positive on the cell membrane and nuclear of tubules cells in normal kidney tissues (Figure 5B), but as for renal cancer samples, the high staining was observed on these cellular structures (Figure 5D). These results, not only corroborate the above findings but also assist clinicians to predict the clinical prognosis of patients.

### 3.7 Biological functions analysis

**FIGURE 6:**
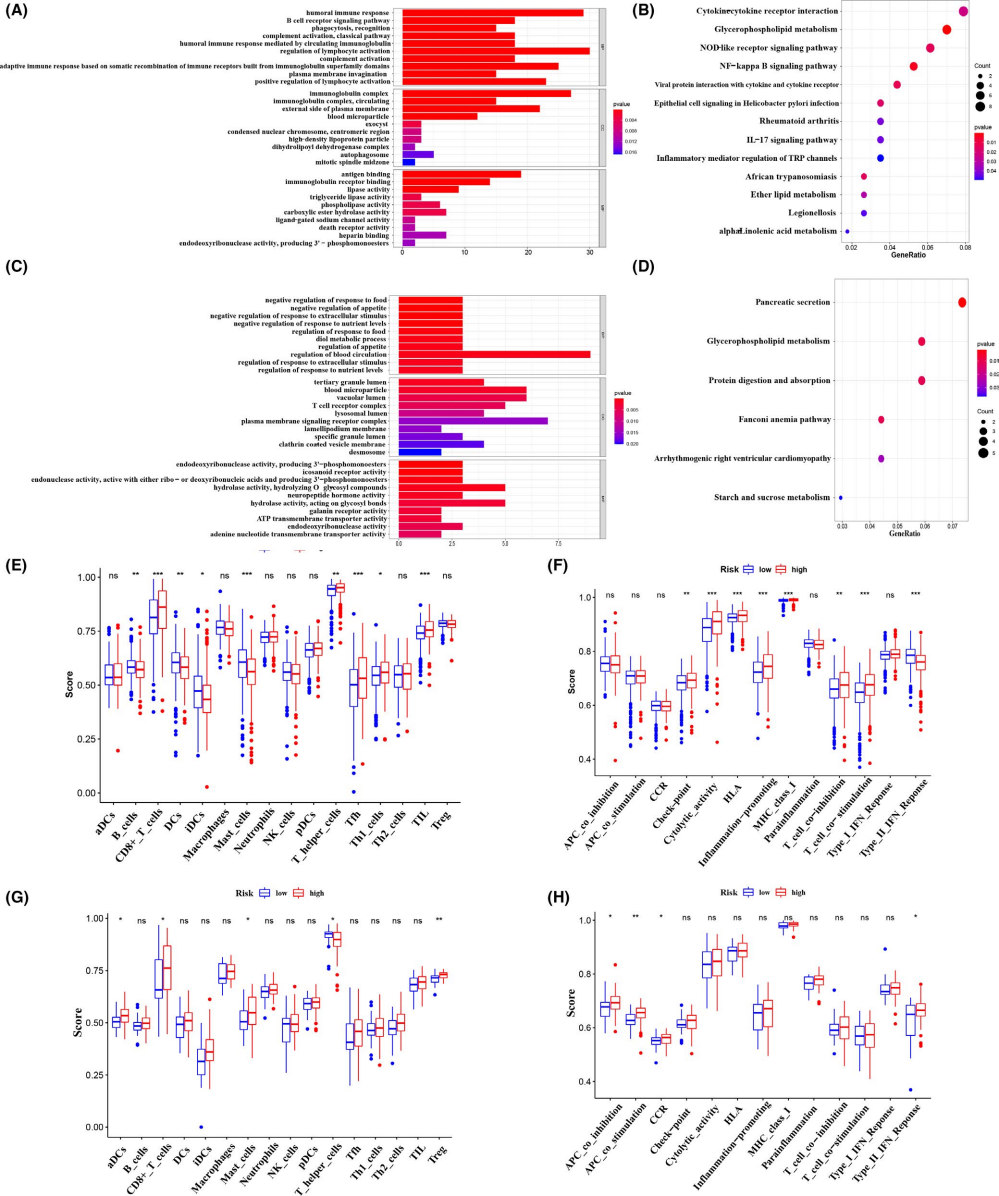
Biological functional analyses in the TCGA and ICGC cohorts. (A, B) Representative results of the GO enrichment (A) and KEGG pathways (B) in TCGA cohort. (C, D) GO enrichment (C) and KEGG pathways (D) in ICGC cohort. (E, F) The ssGSEA scores of 16 immune cells (E) and 13 immune‐related functions (F) between different risk groups in TCGA cohort. (G, H) The ssGSEA scores of 16 immune cells (G) and 13 immune‐related functions (H) in ICGC cohort. *p < 0.05; **p < 0.01; ***p < 0.001

To interrogate the biological functions and pathways behind *HDACs* in TCGA and ICGC cohorts, we performed the GO and KEGG analysis. In TCGA cohort, GO terms are enriched in humoral immune response, positive regulation of lymphocyte activation, regulation of lymphocyte activation, complement activation, classical pathway, B‐cell receptor signaling pathway, and so on (Figure 6A). Moreover, the associated KEGG pathways are enriched in glycerophospholipid metabolism, cytokine−cytokine receptor interaction, NF−kappa B signaling pathway, IL−17 signaling pathway, and so on (Figure 6B). In ICGC ccRCC cohort, GO terms are enriched in lysosomal lumen, T‐cell receptor complex, hydrolase activity, plasma membrane signaling receptor complex, ATP transmembrane transporter activity, and so on (Figure 6C). For KEGG analysis, the associated networks are enriched in glycerophospholipid metabolism, protein digestion and absorption, starch and sucrose metabolism, and so on (Figure 6D).

Then, the GSCAlite database was utilized to figure out the underlying role of *HDACs* in classical pathways, which turned out that *HDACs* may activate or inhibit several oncogenic pathways. For instance, *HDAC11* may activate PI3K/AKT pathway and inhibit apoptosis, cell cycle, and EMT pathways (Figure S10A,B).

Considering the obvious enrichment in various immune‐related processes. We utilized the ssGSEA to calculate enrichment scores of immune cells as well as immune functions. As shown in Figure 6E,F, the enrichment scores of certain immune cells, including CD8^+^ T cell, T helper cell, Th1 cell, Tfh cell, TIL cell, and immune functions such as check‐point, cytolytic activity, and HLA are significantly high in high‐risk set of TCGA ccRCC patients (*p* < 0.05). For ICGC cohort, aDCs cell, mast cells, Treg cells, APC co‐inhibition, APC co‐stimulation have high scores in high‐risk group (*p* < 0.05) (Figure 6G,H).

### 3.8 Correlation analyses of HDAC family in ccRCC

**FIGURE 7:**
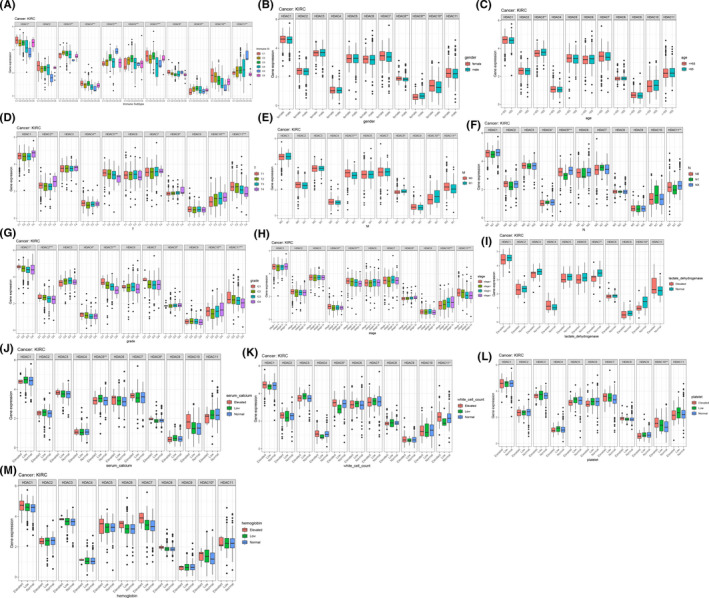
Clinic characteristic and immune subtype correlation analyses. (A) The relationship between HDACs and immune subtypes. (B–M) The correlation between HDACs and clinic features. Box plots show the expression of HDACs. C1: Wound Healing; C2: IFN‐gamma Dominant; C3: Inflammatory; C4: Lymphocyte Deplete; C5: Immunologically Quiet; C6: TGF‐beta Dominant

The immune subtype boxplots (Figure 7A) and clinic correlation boxplots (Figure 7B–M) show the expression within *HDACs* between immune subtypes and clinicopathological features separately. We noticed that *HDAC1* expressed eminently among six immune subtypes, and the expression level in C1 type was obviously higher than other types (*p* < 0.05) (Figure 7A).

The outcomes of the correlation analysis reveal that the expression of some *HDACs* is strongly correlated with clinicopathological characteristics. For example, the expression of *HDAC3*, *HDAC9* are significantly associated with patients’ age and gender separately. Specifically, patients older than 65 or male patients have higher expression level. Moreover, the expression of *HDAC5* and *HDAC11* are positively parallel with tumor stage and grade, T, N, and M stage. Some laboratory markers are also bound up with the expression of *HDACs*. For instance, the expression of *HDAC10* is associated with the level of platelet, lactate dehydrogenase, and hemoglobin (all *p* < 0.05); the expression level of *HDAC5* and *HDAC8* are associated with the level of serum calcium (all *p* < 0.05) and the expression level of *HDAC11* is correlated with the count of white blood cell (*p* < 0.05) (Figure 7B–M).

Next, we scrutinized the relationship between *HDACs* and tumor microenvironment, as well as tumor stemness (Figure S11A). The consequence indicates that most of *HDACs* are linked with tumor stemness, stromal cell, and immune cells. For example, the expression level of *HDAC2* is negatively related to tumor stemness (data based on RNA expression) (*p* = 0.035), and the higher the expression level of *HDAC2*, the more stromal cells (*p* < 0.001) instead of immune cells in the tumor microenvironment. In addition, given several genes mutations have been described in ccRCC, we also conducted the correlation analysis between *HDAC8*, *HDAC10*, *HDAC11,* and common mutated genes, including *VHL*, *PBRM1*, *mTOR,* and *BAP1* ^15^ (Figure S12,A‐I). The result indicates that all HDACs are positively correlated with common mutated genes, except that HDAC10 has no correlation with *PBRM1* (Figure S12E).

Finally, the correlation analysis of all *HDACs* and drug activity was analyzed using data from the CellMiner database (Table S1; Figure S11B). The top 16 relevant correlations between *HDACs* and drug activity are shown in Figure S11B. We found that the activity of some commonly used drugs is negatively related to the expression level of *HDAC*11, including oxaliplatin, carmustine, ifosfamide, lmexon, lomustine, and BN‐2629. In addition, the activity of some common drugs has a positive relationship between *HDACs* expression as well. By way of illustration, the sensitivity of temsirolimus, a specific inhibitor of mTOR and HDAC inhibitor vorinostat are positively correlated with *HDAC*10 expression (*r* = 0.315, *p* = 0.014).

## 4 DISCUSSION

With the huge revolution and decreased cost of gene sequencing, a vast amount of genetic information is easily accessible to researchers.^16^ However, most of the precision treatments targeting at gene alterations are seldom available. At the same time, under the huge pressure of high cost, only a limited percentage of patients could benefit from precise treatments.^17^, ^18^ Nowadays, faced with a limited understanding of the biological relationship between tumor genotype and phenotype, some pivotal molecular signatures need to be explored.

It is reported that histone modification, such as H3Ac, H4Ac, H3K18Ac, and H3K27me3, plays a pivotal role in tumorigenesis and progression.^8^, ^19^ Histone acetylation, as a common type of histone modifications, involves many enzymes that could add or remove acetylation markers,^6^ which implies that histone acetylation may serve as a potential target for tumor therapy. Recent literature has demonstrated that inhibiting histone deacetylase (especially *HDAC2*) could reverse drug resistance to angiogenesis inhibitors in RCC patients.^20^ *HDAC8* is a multifaceted target for therapeutic interventions in colon, lung, and hepatocellular carcinoma cervical cancers as well, which regulates proliferation and apoptosis in cancer cells.^21^, ^22^ *HDAC10* were also found to be prognostic markers for gastric cancer and colon cancer.^23^, ^24^ Reports in PNAS and Autophagy illustrate that *HDAC10* could promote autophagy‐mediated survival in neuroblastoma and improve treatment response of advanced neuroblastomas.^25^, ^26^ In lung cancer, *HDAC10* is positively associated with the expression level of PD‐L1, which acts as an independent prognostic factor^27^ and regulates stem‐like lung adenocarcinoma cell.^28^ Fan W et al^29^ found that *HDAC10* expression was suppressed in ccRCC and also involved in the development and metastasis of ccRCC. Moreover, prior research noted that *HDAC11* was a novel prognostic marker, affecting apoptosis and maintaining the metabolism and viability of cancer cells in prostate cancer, pancreatic cancer, ovarian and breast cancer.^30^, ^31^ As few researchers focus on other members of *HDAC* family, their roles in ccRCC are still far from being known and require further investigation.

We explored here the expression profile of *HDAC* family via Oncomine database, which indicated that *HDAC1*, *HDAC2*, *HDAC3*, *HDAC7*, *HDAC8,* and *HDAC9* expressed highly in pan‐cancer at transcriptional level. Then, the same trend was observed by using TCGA RNA‐seq data. We also utilized the CTPAC protein expression data from UALCAN online tools to analyze *HDACs* expression profile on protein level, and the results were the same as the RNA transcriptional data as well. Considering the key role of tumor mutations in regulating anticancer immunity,^32^ we analyzed the SNV and CNV of *HDACs* in pan‐cancers by utilizing the GSCAlite database. The results turned out that most of HDACs mutated at pan‐cancer, and missense mutation occupied the most part in all types of mutations. Then, we exploit overall survival and identified that low expression level of *HDAC1*, *HDAC2*, *HDAC3*, *HDAC8*, *HDAC10,* and high expression level of *HDAC5*, *HDAC7*, and *HDAC11* had better survival in RCC. To deeply explore the prognostic value of HDAC family in ccRCC, we filtered out *HDAC8*, *HDAC10,* and *HDAC11* and constructed a risk signature after univariate Cox analysis and LASSO regression analysis by data from the TCGA database. The results from K–M curves, risk score profiles, and multivariate Cox analysis all illustrated a favorable role in risk prediction. In our validation cohort, including patients in the ICGC database and HPA database, we still observed a significantly differential survival trend for the risk model, which suggested the accurate efficiency of the risk model consisting of *HDAC8*, *HDAC10,* and *HDAC11*. The underlying mechanisms were interpreted by GO analysis, KEGG analysis, ssGSEA analysis, and correlation analyses between *HDACs* and immune subtypes and tumor stemness. The results presented that immune‐associated functions and pathways were enriched in *HDACs*, and most of *HDACs* are related to tumor stemness, stromal cell, and immune cells, which is consistent with previous studies.^33^, ^34^, ^35^ Besides, further validation from the GSCAlite database demonstrated that *HDACs* are involved in PI3K/AKT pathway and EMT pathways. Finally, for the assessment of clinical value, we validated the relationship between *HDACs* and clinicopathological characteristics, as well as drug activity. Our finding indicated that patients’ age and gender, tumor stage, and grade, T, N, and M stage, and laboratory markers (platelet, lactate dehydrogenase, serum calcium, white blood cell count, and hemoglobin) were significantly associated with *HDACs* expression. Noticeably, the activity of some commonly used drugs (such as oxaliplatin, vorinostat, temsirolimus) is also influenced by *HDACs*.

Nowadays, with the first approval of vorinostat (a HDAC inhibitor) by FDA, more HDAC inhibitors have been used to treat malignant tumors. In RCC, using vorinostat alone or combining vorinostat and temsirolimus inhibited the proliferation and angiogenesis in vitro and vivo models.^36^, ^37^ Moreover, the combination of HDAC inhibitor valproic acid and everolimus may hinder drug resistance caused by long‐term everolimus treatment.^38^ However, the majority of HDAC inhibitors were applied to hematological tumors, and unfortunately the demonstrated effect in solid tumors is not as effective as hematological tumors.^39^ Therefore, further exploration of HDAC biological functions and rapid development of potent‐specific inhibitors is of the essence.

To improve the outcome of ccRCC and the effect of HDAC inhibitor, it is necessary to identify ccRCC patients who could benefit the most from treatments at the first diagnosis. Therefore, accurate and efficient biomarkers are indispensable. Our study purposed to reveal the molecular mechanism as well as clinical value, and the results suggest *HDAC8*, *HDAC10,* and *HDAC11* could be used to estimate patients’ prognosis and serve as potential therapeutic targets. In future investigations, patients’ data from our center will be collected to valid this signature and further experiments in vivo and vitro will be implemented to confirm the possibility as prognostic biomarkers as well.

## CONFLICT OF INTEREST

None.

## AUTHOR CONTRIBUTIONS

B.Z. and W.H. involved in conceptualization; B.Z., F.J.C., Z.S.Y., and G.T.Z. involved in methodology; B.Z. and J.W.W. carried out software preparation; F.J.C., B.Z. also carried out writing of the manuscript; and Z.H.N., W.H. involved in project administration.

## ETHICAL APPROVAL STATEMENT

None.

## Supporting information

Fig S1

Fig S2

Fig S3

Fig S4

Fig S5

Fig S6

Fig S7

Fig S8

Fig S9

Fig S10

Fig S11

Fig S12

Table S1

## Data Availability

The data that support the findings are available from the TCGA and ICGA databases.
