# Supplementary figures and images for: Comprehensive analysis of a new prognosis signature based on histone deacetylases in clear cell renal cell carcinoma
Source: Cancer Med. 2021 Jul 26;10(18):6503–14. doi: 10.1002/cam4.4156 (PMC8446567; doi:10.1002/cam4.4156)

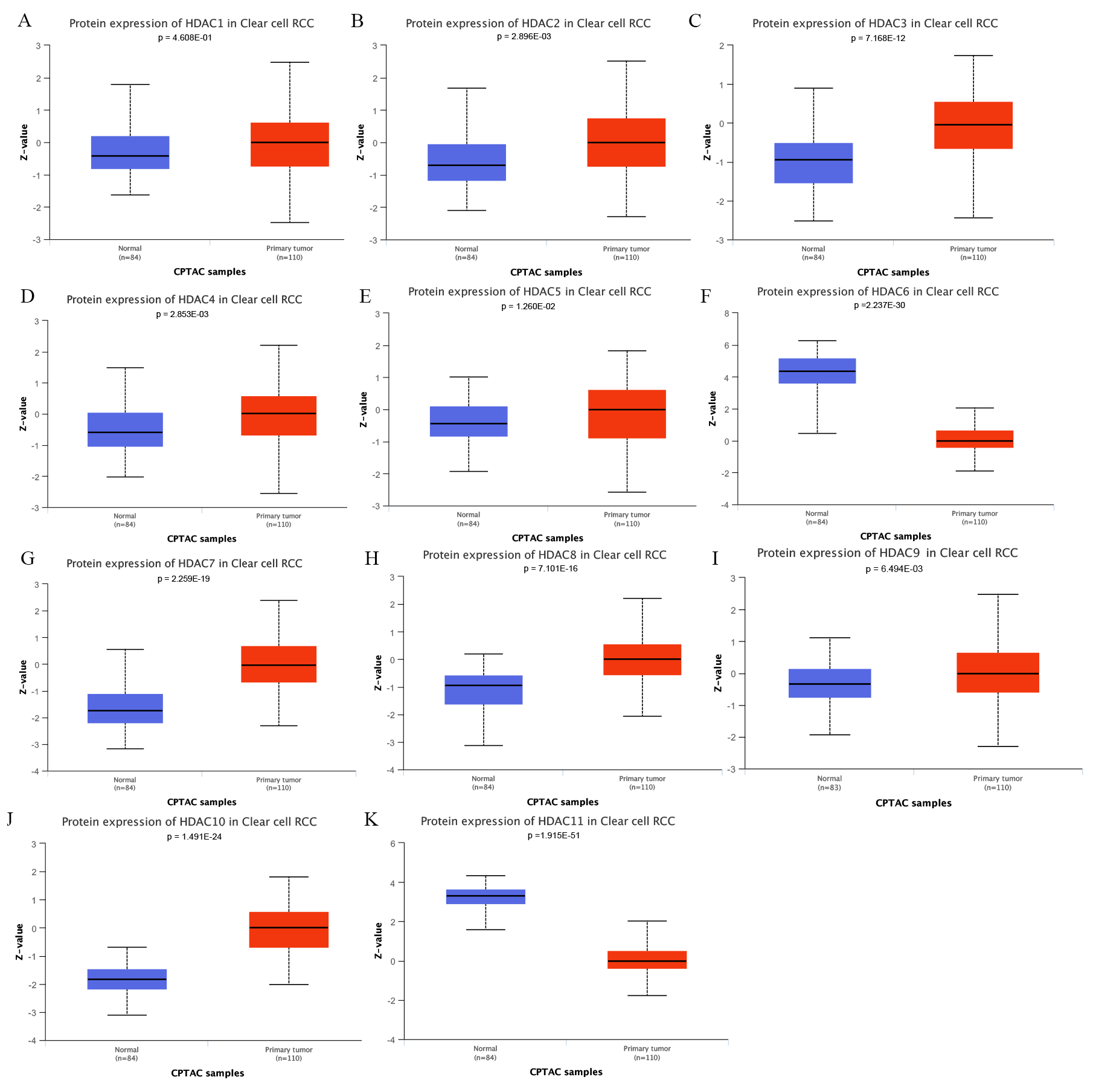

Supplement: Supplementary file 1 — Fig S1 [file CAM4-10-6503-s001.tif]

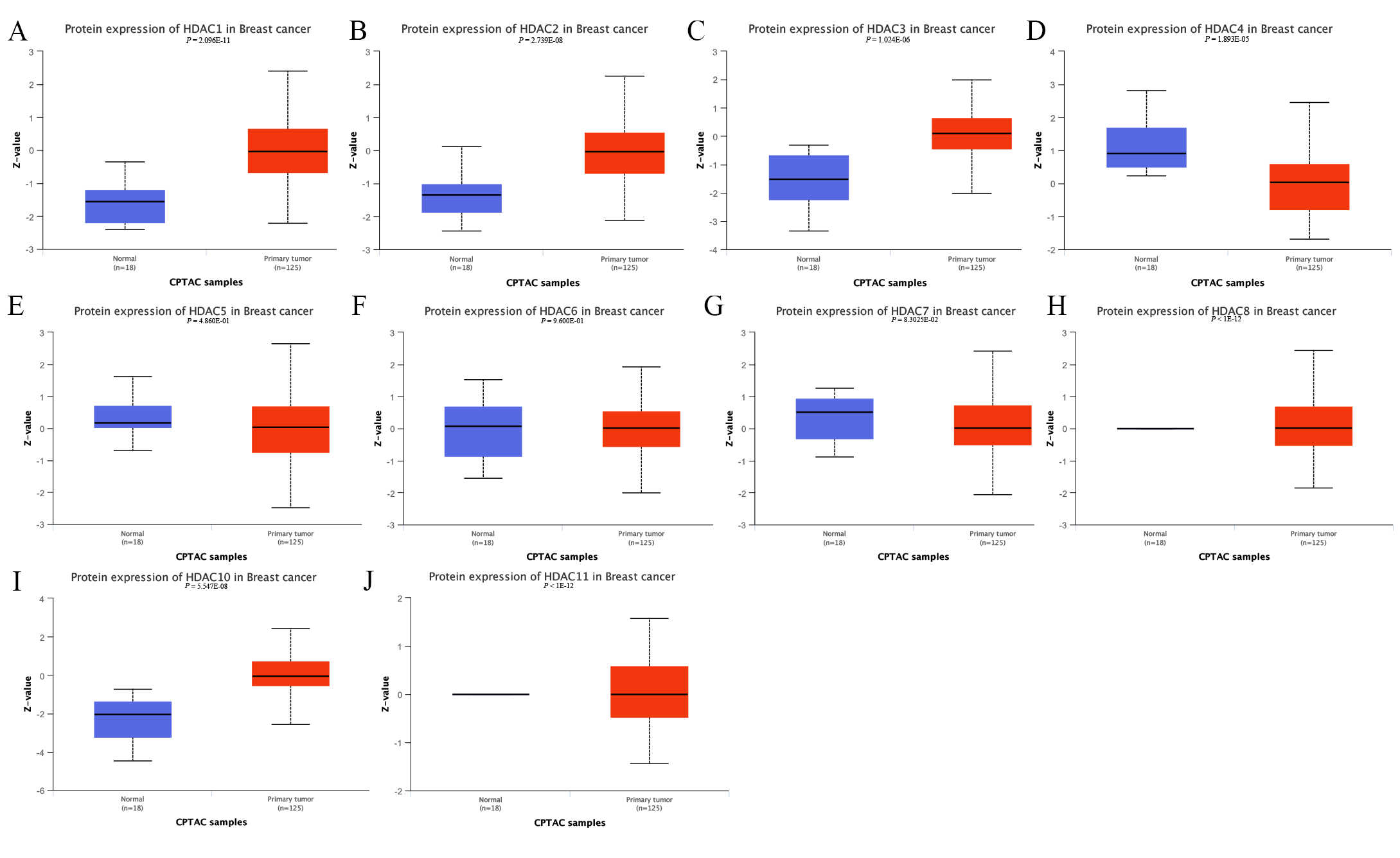

Supplement: Supplementary file 2 — Fig S2 [file CAM4-10-6503-s008.tif]

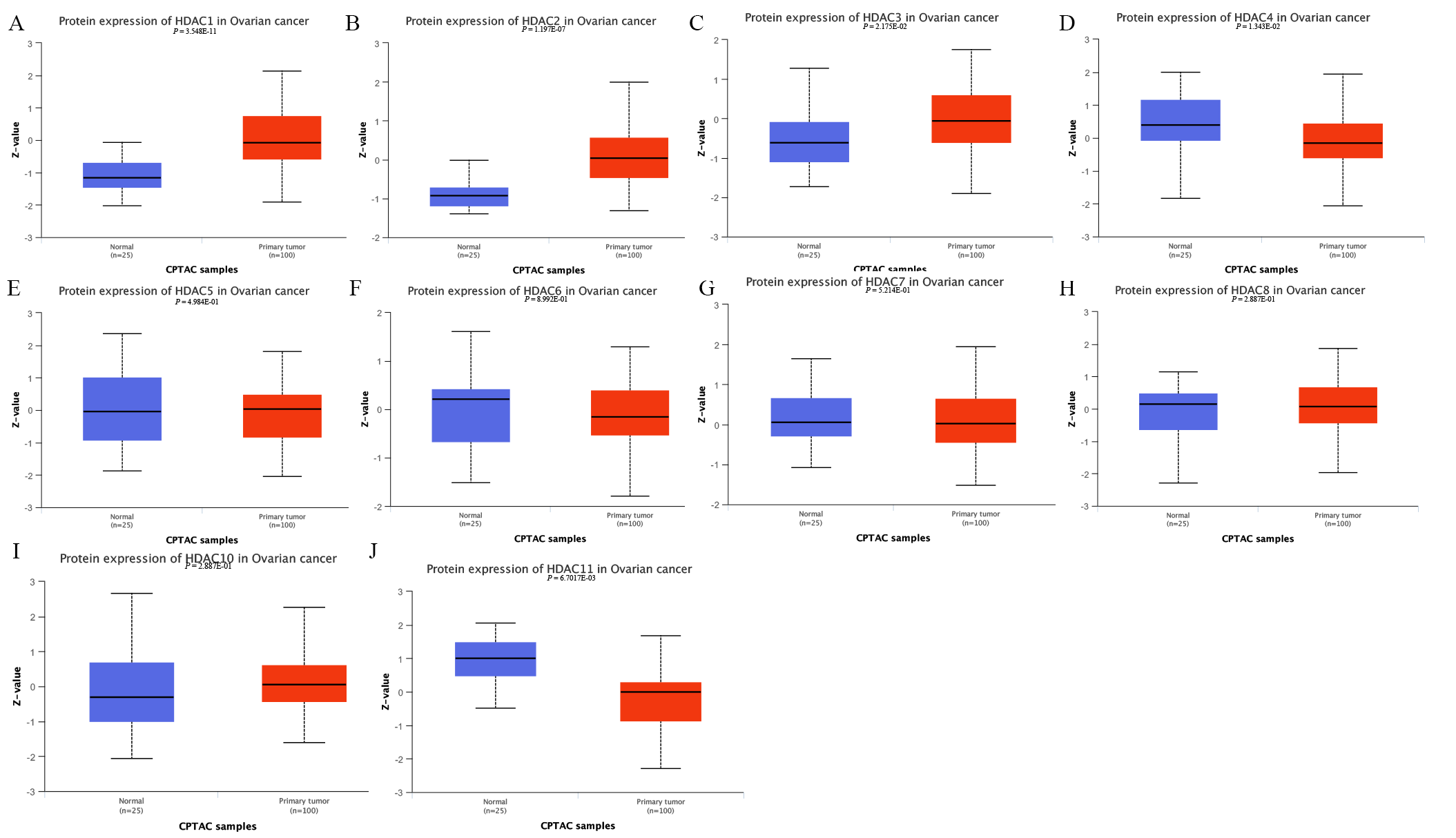

Supplement: Supplementary file 3 — Fig S3 [file CAM4-10-6503-s013.tif]

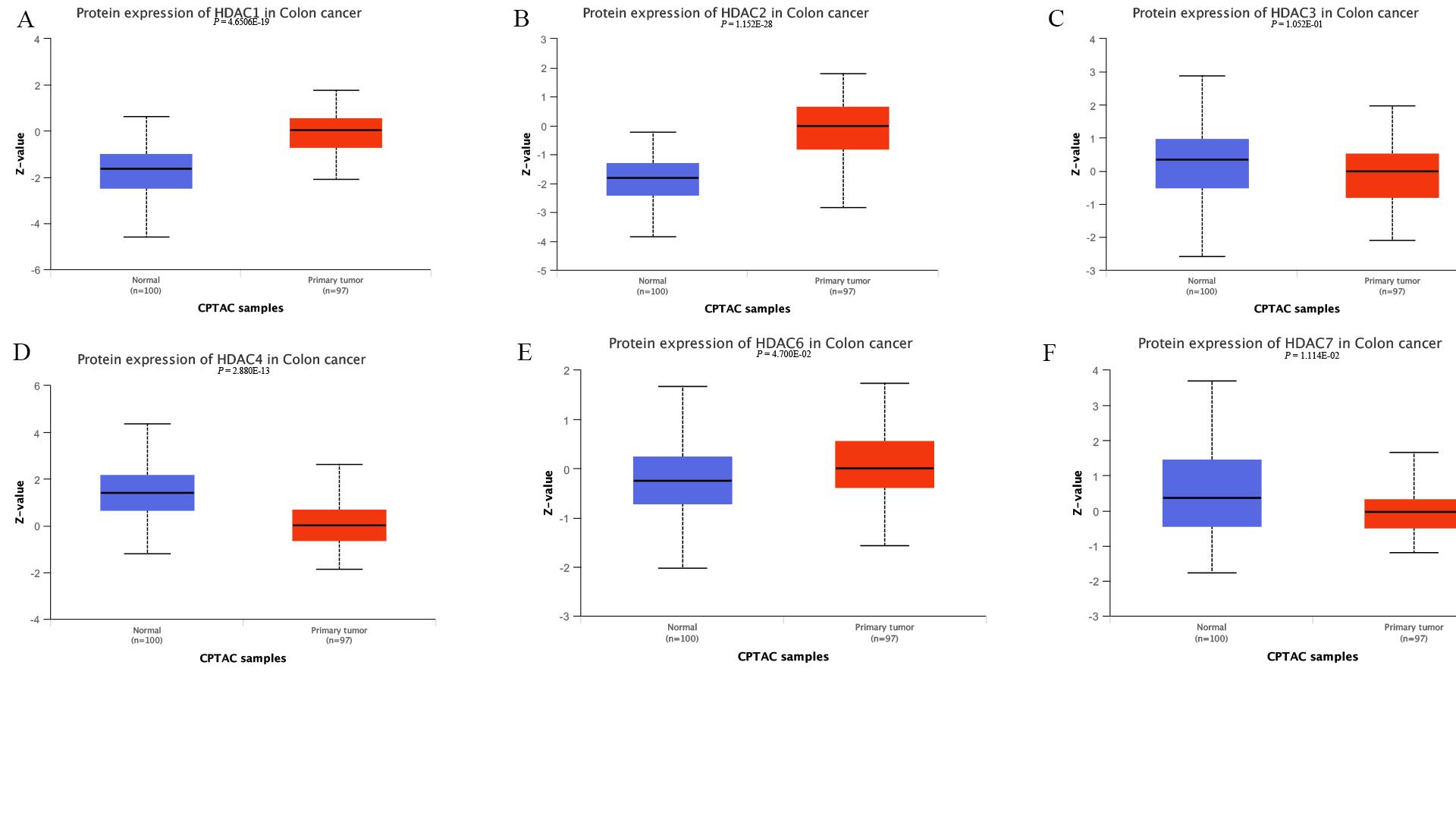

Supplement: Supplementary file 4 — Fig S4 [file CAM4-10-6503-s004.tif]

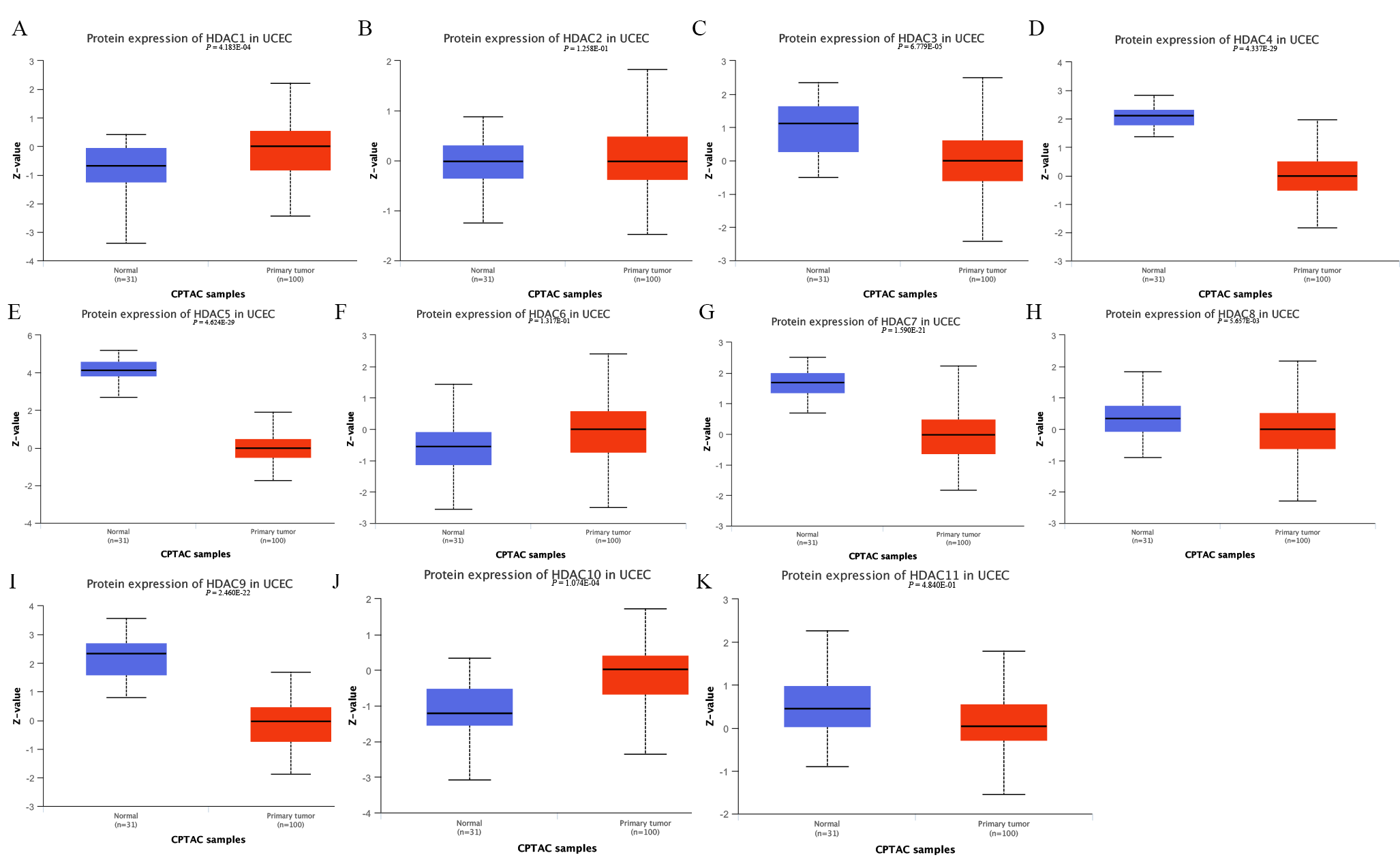

Supplement: Supplementary file 5 — Fig S5 [file CAM4-10-6503-s006.tif]

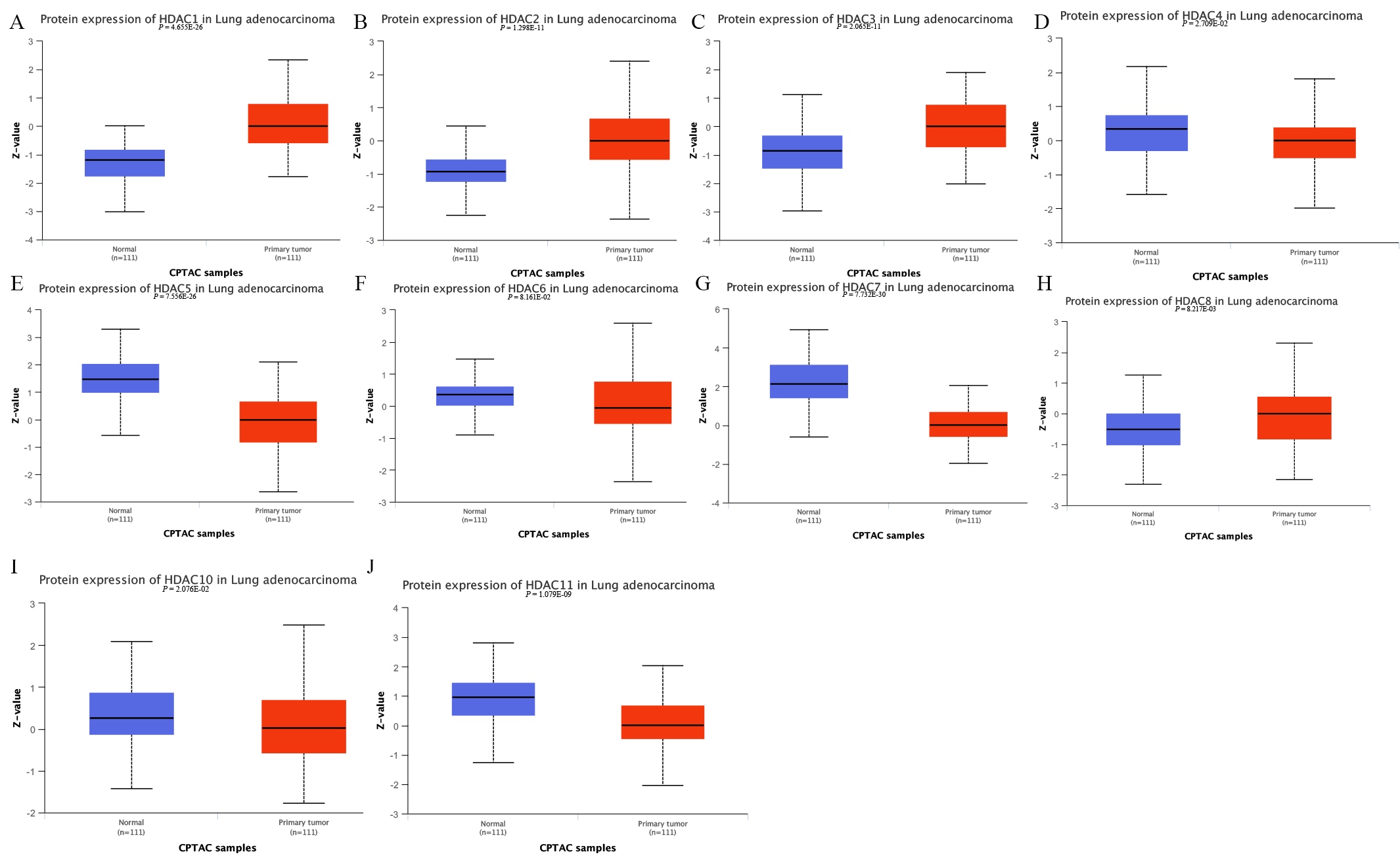

Supplement: Supplementary file 6 — Fig S6 [file CAM4-10-6503-s011.tif]

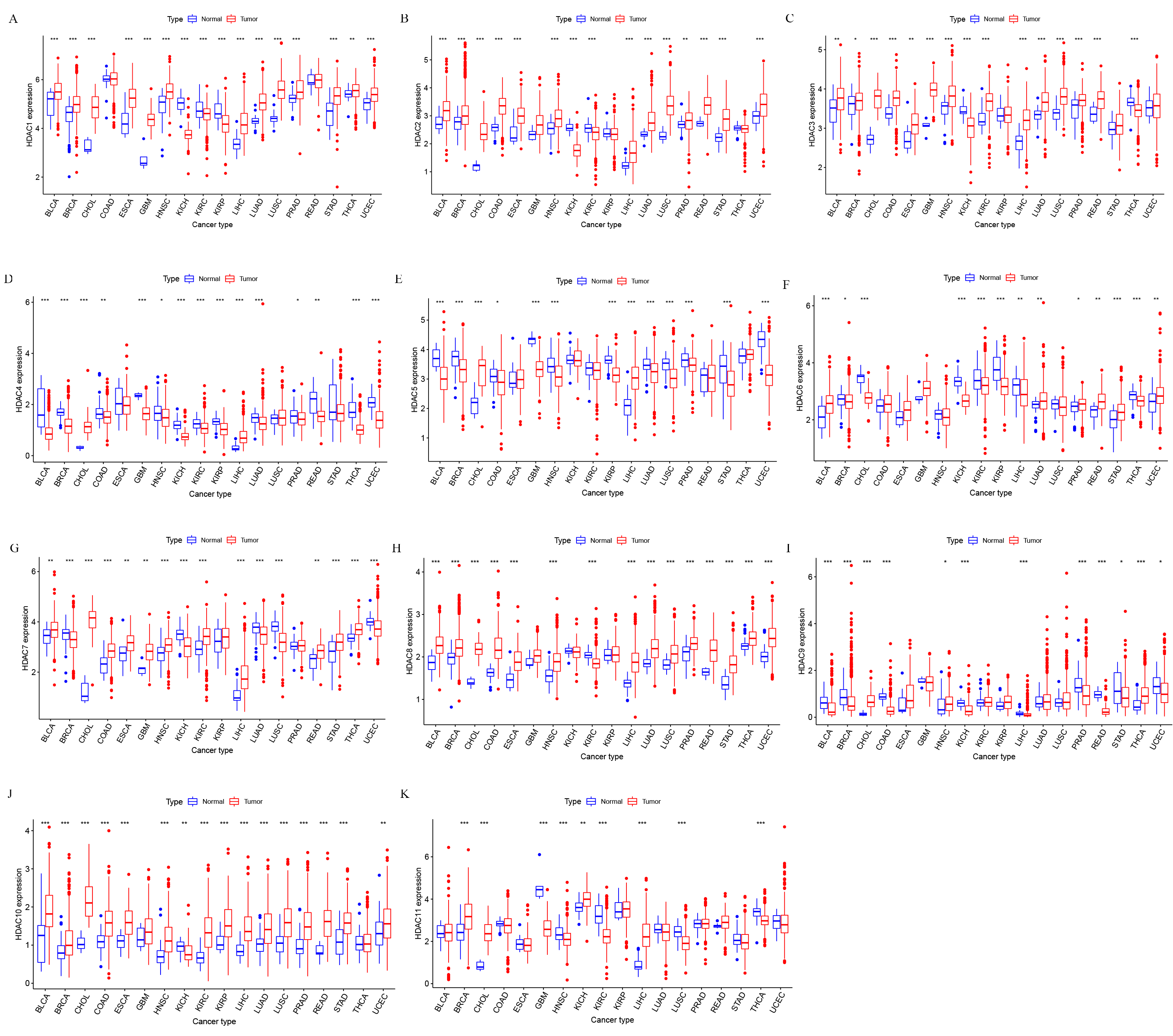

Supplement: Supplementary file 7 — Fig S7 [file CAM4-10-6503-s003.tif]

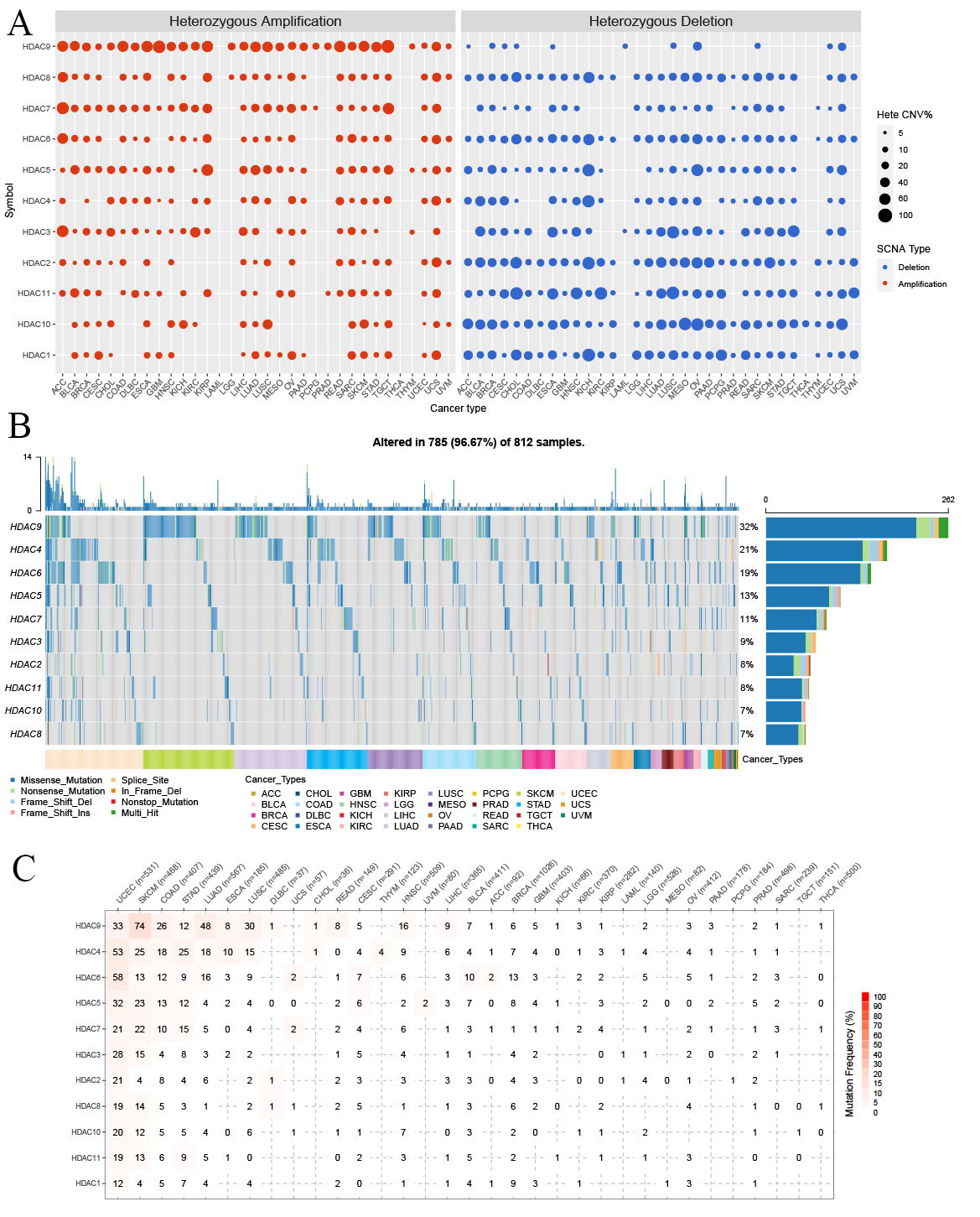

Supplement: Supplementary file 8 — Fig S8 [file CAM4-10-6503-s002.tif]

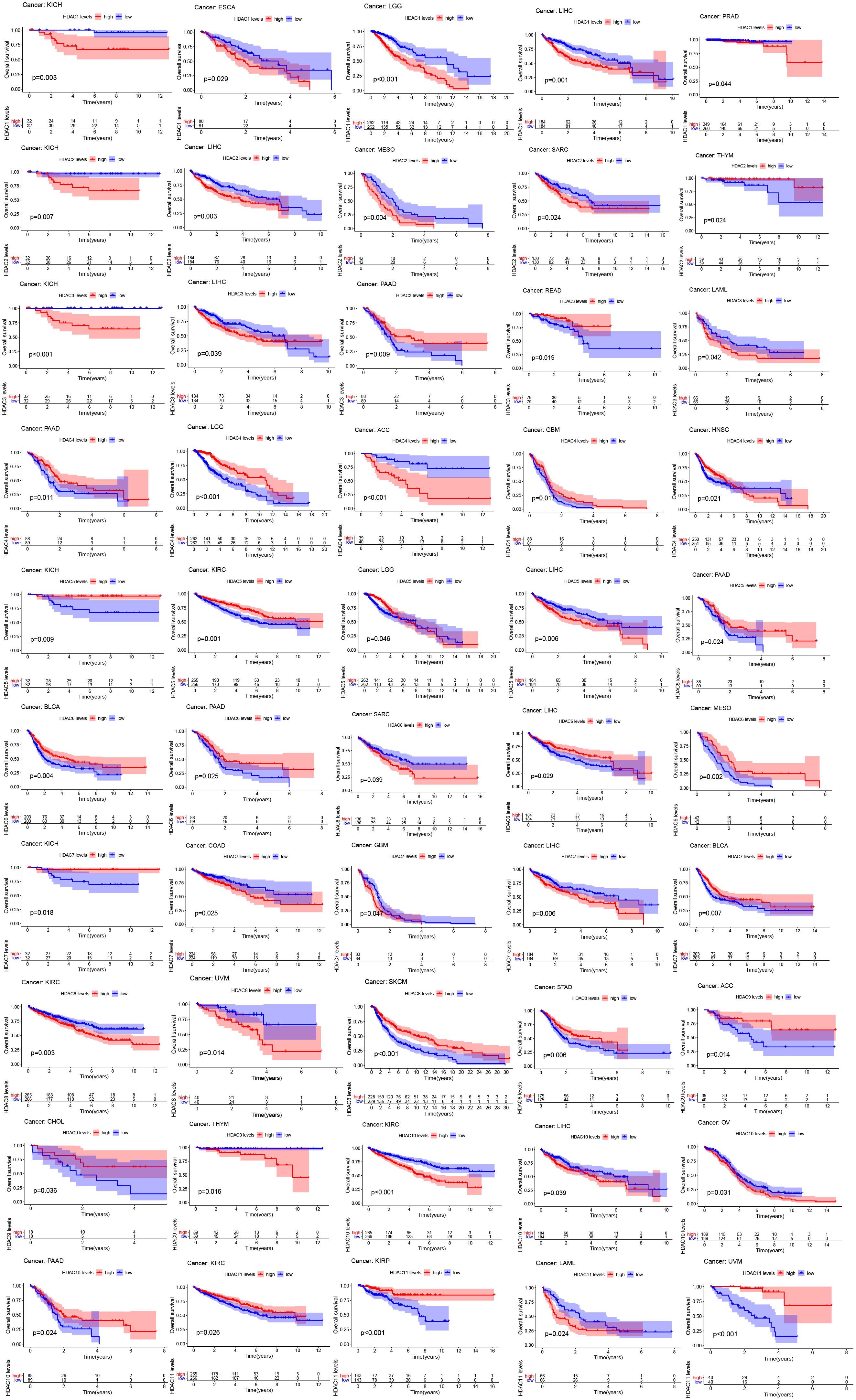

Supplement: Supplementary file 9 — Fig S9 [file CAM4-10-6503-s009.tif]

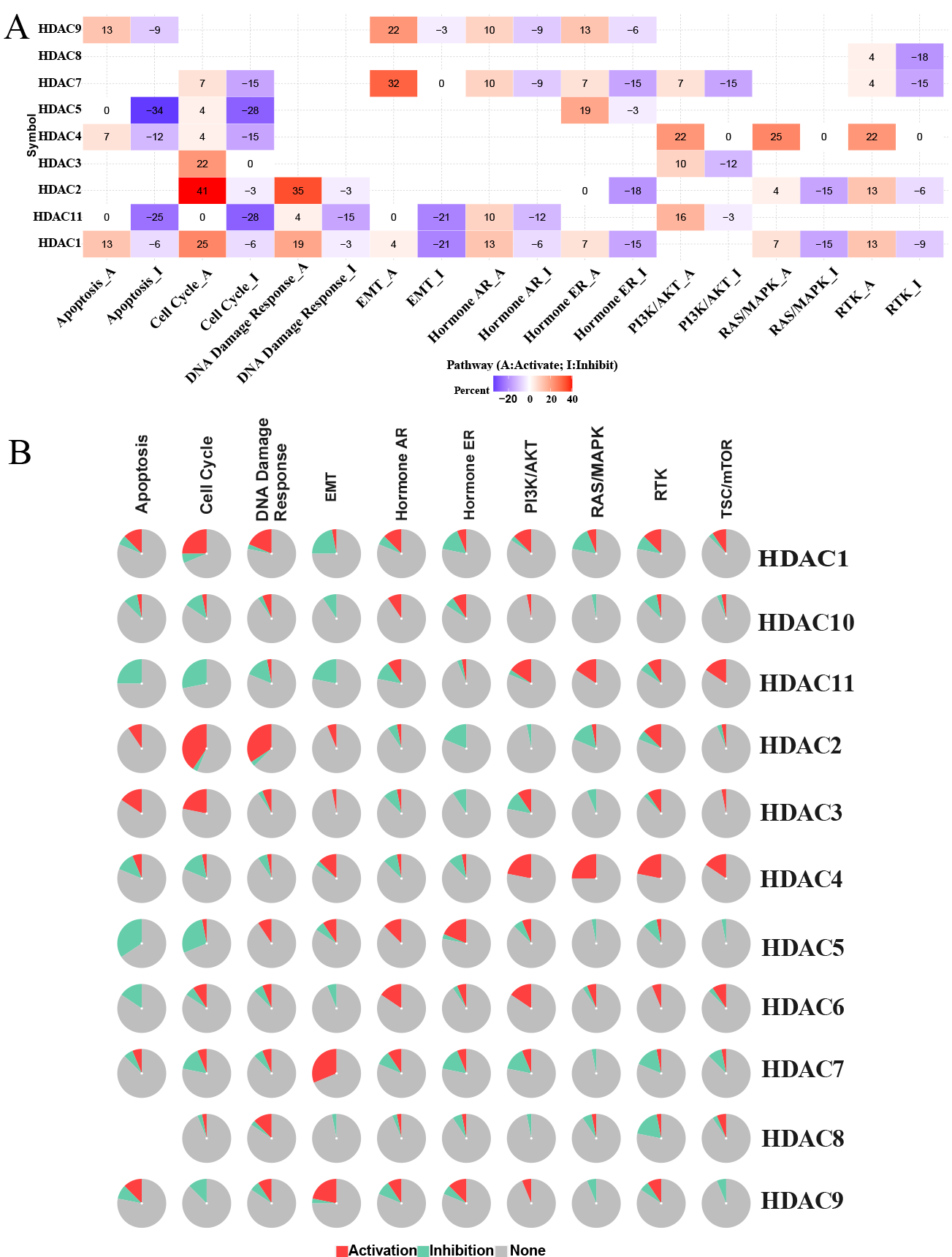

Supplement: Supplementary file 10 — Fig S10 [file CAM4-10-6503-s007.tif]

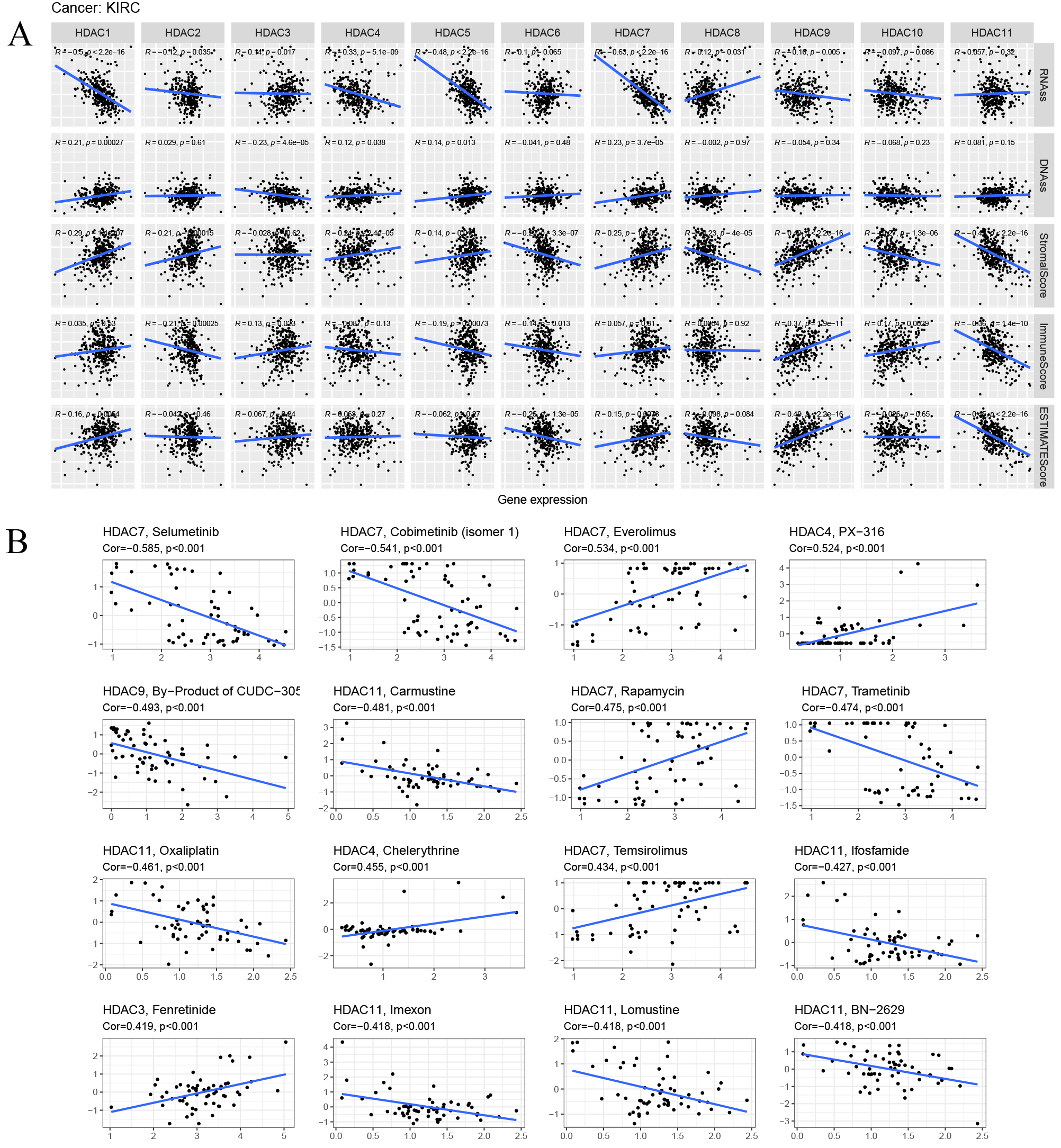

Supplement: Supplementary file 11 — Fig S11 [file CAM4-10-6503-s005.tif]

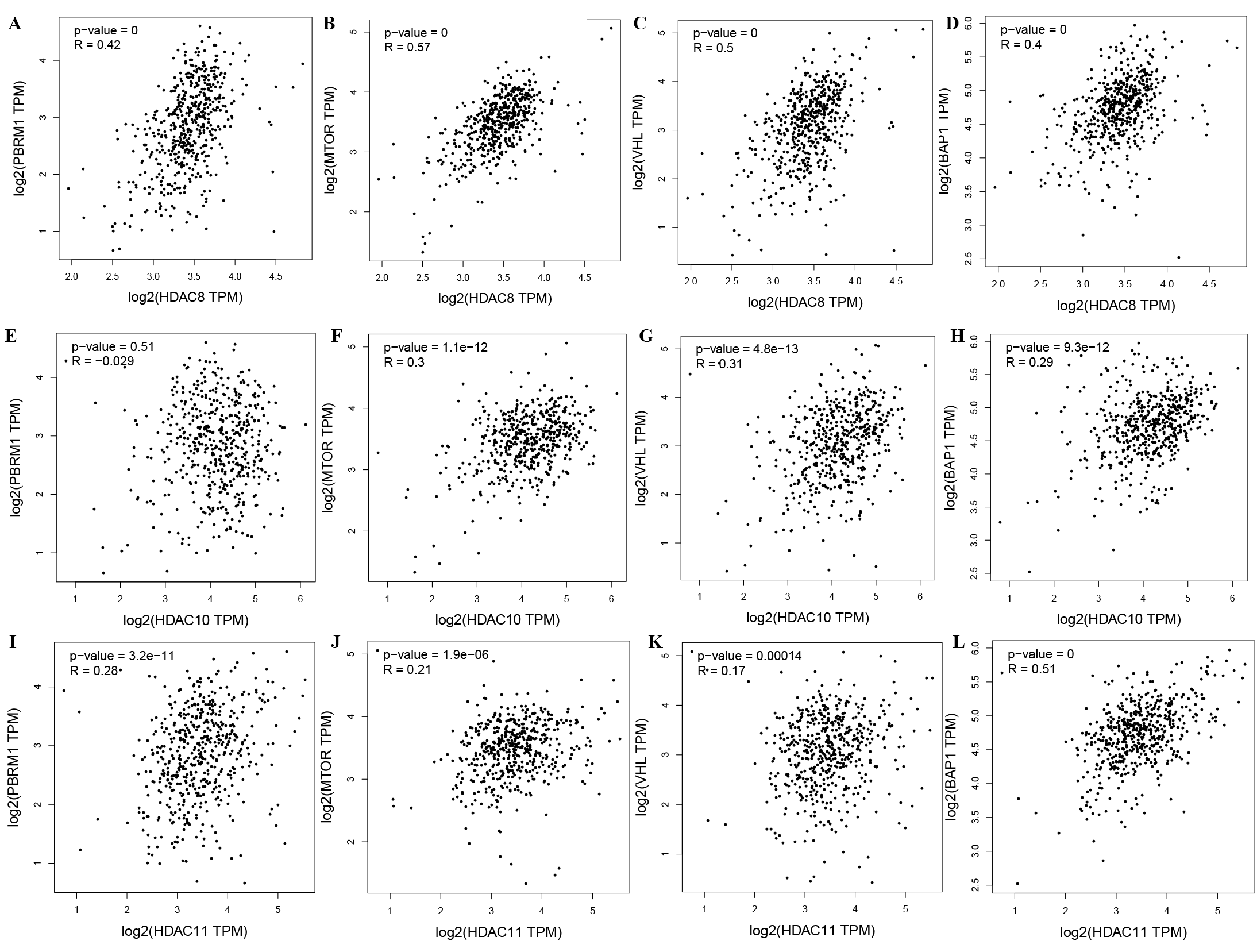

Supplement: Supplementary file 12 — Fig S12 [file CAM4-10-6503-s010.tif]
