# Supplementary material for: Comprehensive analysis of a new prognosis signature based on histone deacetylases in clear cell renal cell carcinoma
Source: Cancer Med. 2021 Jul 26;10(18):6503–14. doi: 10.1002/cam4.4156 (PMC8446567; doi:10.1002/cam4.4156)
Supplement: Supplementary file 13 — Table S1 [file CAM4-10-6503-s012.docx]

**Supplementary Table1. The correlation between drug activity and HDACs expression level**

| Gene | Drug | cor | *p*-value |
| --- | --- | --- | --- |
| HDAC1 | Chelerythrine | 0.409 | 0.001 |
| HDAC1 | Acrichine | 0.406 | 0.001 |
| HDAC1 | Nelarabine | 0.400 | 0.002 |
| HDAC1 | Fenretinide | 0.398 | 0.002 |
| HDAC1 | Pyrazoloacridine | 0.366 | 0.004 |
| HDAC1 | Perifosine | 0.353 | 0.006 |
| HDAC1 | 8-Chloro-adenosine | 0.338 | 0.008 |
| HDAC1 | PX-316 | 0.335 | 0.009 |
| HDAC1 | Fostamatinib | 0.334 | 0.009 |
| HDAC1 | Curcumin | 0.309 | 0.016 |
| HDAC1 | Sunitinib | 0.305 | 0.018 |
| HDAC1 | Erlotinib | -0.282 | 0.029 |
| HDAC1 | RH1 | 0.276 | 0.033 |
| HDAC1 | Parthenolide | 0.273 | 0.035 |
| HDAC1 | Vismodegib | 0.269 | 0.037 |
| HDAC1 | Hydroxyurea | 0.268 | 0.039 |
| HDAC1 | LY-294002 | -0.264 | 0.041 |
| HDAC1 | 7-Hydroxystaurosporine | 0.264 | 0.042 |
| HDAC1 | Palbociclib | 0.261 | 0.044 |
| HDAC1 | Hydrastinine HCl | -0.259 | 0.046 |
| HDAC1 | Methotrexate | 0.258 | 0.047 |
| HDAC1 | Cladribine | 0.257 | 0.048 |
| HDAC1 | Crizotinib | 0.255 | 0.049 |
| HDAC1 | Vandetanib | -0.254 | 0.050 |
| HDAC2 | Chelerythrine | 0.333 | 0.009 |
| HDAC2 | PX-316 | 0.330 | 0.010 |
| HDAC2 | Ifosfamide | 0.300 | 0.020 |
| HDAC2 | Pyrazoloacridine | 0.297 | 0.021 |
| HDAC2 | Nelarabine | 0.289 | 0.025 |
| HDAC2 | O-6-Benzylguanine | -0.260 | 0.045 |
| HDAC2 | Everolimus | -0.256 | 0.048 |
| HDAC3 | Fenretinide | 0.419 | 0.001 |
| HDAC3 | Nelarabine | 0.380 | 0.003 |
| HDAC3 | 5-fluoro deoxy uridine 10mer | 0.350 | 0.006 |
| HDAC3 | Denileukin Diftitox Ontak | -0.335 | 0.009 |
| HDAC3 | Cladribine | 0.330 | 0.010 |
| HDAC3 | Chelerythrine | 0.322 | 0.012 |
| HDAC3 | 7-Ethyl-10-hydroxycamptothecin | 0.319 | 0.013 |
| HDAC3 | Floxuridine | 0.314 | 0.015 |
| HDAC3 | Triethylenemelamine | 0.296 | 0.021 |
| HDAC3 | Fludarabine | 0.293 | 0.023 |
| HDAC3 | Chlorambucil | 0.291 | 0.024 |
| HDAC3 | Pemetrexed | 0.285 | 0.027 |
| HDAC3 | Axitinib | 0.282 | 0.029 |
| HDAC3 | Selumetinib | -0.281 | 0.030 |
| HDAC3 | Hydroxyurea | 0.273 | 0.035 |
| HDAC3 | Thiotepa | 0.272 | 0.036 |
| HDAC3 | Gemcitabine | 0.271 | 0.036 |
| HDAC3 | Clofarabine | 0.271 | 0.036 |
| HDAC3 | Cobimetinib (isomer 1) | -0.271 | 0.037 |
| HDAC3 | Irinotecan | 0.266 | 0.040 |
| HDAC3 | Cytarabine | 0.265 | 0.041 |
| HDAC3 | Uracil mustard | 0.265 | 0.041 |
| HDAC3 | Topotecan | 0.260 | 0.045 |
| HDAC3 | tfdu | 0.258 | 0.047 |
| HDAC4 | PX-316 | 0.524 | 0.000 |
| HDAC4 | Chelerythrine | 0.455 | 0.000 |
| HDAC4 | Selumetinib | 0.404 | 0.001 |
| HDAC4 | Dasatinib | -0.401 | 0.002 |
| HDAC4 | Nelarabine | 0.386 | 0.002 |
| HDAC4 | LY-294002 | -0.376 | 0.003 |
| HDAC4 | Everolimus | -0.353 | 0.006 |
| HDAC4 | Hypothemycin | 0.350 | 0.006 |
| HDAC4 | PD-98059 | 0.339 | 0.008 |
| HDAC4 | Dexrazoxane | 0.333 | 0.009 |
| HDAC4 | XK-469 | 0.333 | 0.009 |
| HDAC4 | Batracylin | 0.322 | 0.012 |
| HDAC4 | Acetalax | -0.321 | 0.012 |
| HDAC4 | Ifosfamide | 0.317 | 0.013 |
| HDAC4 | Azacitidine | -0.306 | 0.017 |
| HDAC4 | Asparaginase | 0.304 | 0.018 |
| HDAC4 | Alvespimycin | 0.302 | 0.019 |
| HDAC4 | Acrichine | 0.297 | 0.021 |
| HDAC4 | Vemurafenib | 0.293 | 0.023 |
| HDAC4 | Cobimetinib (isomer 1) | 0.292 | 0.024 |
| HDAC4 | bisacodyl, active ingredient of viraplex | -0.292 | 0.024 |
| HDAC4 | Erlotinib | -0.290 | 0.025 |
| HDAC4 | Seliciclib | -0.287 | 0.026 |
| HDAC4 | Sonidegib | -0.286 | 0.026 |
| HDAC4 | Zoledronate | -0.284 | 0.028 |
| HDAC4 | Bendamustine | 0.277 | 0.032 |
| HDAC4 | Trametinib | 0.275 | 0.034 |
| HDAC4 | Itraconazole | -0.274 | 0.034 |
| HDAC4 | Lenvatinib | -0.270 | 0.037 |
| HDAC4 | Dabrafenib | 0.267 | 0.039 |
| HDAC4 | Pipamperone | 0.259 | 0.046 |
| HDAC5 | Palbociclib | -0.327 | 0.011 |
| HDAC5 | Seliciclib | 0.315 | 0.014 |
| HDAC5 | Dexrazoxane | -0.306 | 0.017 |
| HDAC5 | Curcumin | 0.300 | 0.020 |
| HDAC5 | XL-147 | 0.294 | 0.022 |
| HDAC5 | Ethinyl estradiol | 0.272 | 0.036 |
| HDAC5 | Vorinostat | 0.269 | 0.038 |
| HDAC5 | Depsipeptide | -0.264 | 0.042 |
| HDAC6 | Lificguat | -0.341 | 0.008 |
| HDAC6 | AFP464 | -0.334 | 0.009 |
| HDAC6 | Fludarabine | 0.297 | 0.021 |
| HDAC6 | Testolactone | 0.290 | 0.025 |
| HDAC6 | Aminoflavone | -0.283 | 0.028 |
| HDAC6 | Nelarabine | 0.267 | 0.039 |
| HDAC7 | Selumetinib | -0.585 | 0.000 |
| HDAC7 | Cobimetinib (isomer 1) | -0.541 | 0.000 |
| HDAC7 | Everolimus | 0.534 | 0.000 |
| HDAC7 | Rapamycin | 0.475 | 0.000 |
| HDAC7 | Trametinib | -0.474 | 0.000 |
| HDAC7 | Temsirolimus | 0.434 | 0.001 |
| HDAC7 | PD-98059 | -0.407 | 0.001 |
| HDAC7 | Dabrafenib | -0.402 | 0.001 |
| HDAC7 | Dolastatin 10 | -0.402 | 0.001 |
| HDAC7 | Vemurafenib | -0.397 | 0.002 |
| HDAC7 | okadaic acid | -0.368 | 0.004 |
| HDAC7 | Tanespimycin | -0.368 | 0.004 |
| HDAC7 | ABT-199 | -0.365 | 0.004 |
| HDAC7 | Paclitaxel | -0.361 | 0.005 |
| HDAC7 | Triciribine phosphate | 0.355 | 0.005 |
| HDAC7 | Bafetinib | -0.354 | 0.006 |
| HDAC7 | geldanamycin analog | -0.348 | 0.006 |
| HDAC7 | Hypothemycin | -0.345 | 0.007 |
| HDAC7 | Depsipeptide | -0.332 | 0.010 |
| HDAC7 | Vinblastine | -0.327 | 0.011 |
| HDAC7 | Ibrutinib | 0.321 | 0.012 |
| HDAC7 | Erlotinib | 0.313 | 0.015 |
| HDAC7 | Actinomycin D | -0.304 | 0.018 |
| HDAC7 | Dasatinib | 0.298 | 0.021 |
| HDAC7 | Bleomycin | 0.295 | 0.022 |
| HDAC7 | Tamoxifen | -0.278 | 0.031 |
| HDAC7 | Tyrothricin | -0.267 | 0.039 |
| HDAC7 | Vinorelbine | -0.265 | 0.040 |
| HDAC7 | Nelarabine | 0.265 | 0.040 |
| HDAC7 | 6-Mercaptopurine | 0.259 | 0.046 |
| HDAC7 | Eribulin mesilate | -0.255 | 0.049 |
| HDAC8 | PX-316 | 0.336 | 0.009 |
| HDAC8 | bisacodyl, active ingredient of viraplex | -0.332 | 0.010 |
| HDAC8 | Acetalax | -0.304 | 0.018 |
| HDAC8 | Ethinyl estradiol | 0.290 | 0.024 |
| HDAC8 | Ifosfamide | 0.289 | 0.025 |
| HDAC8 | Fenretinide | 0.283 | 0.028 |
| HDAC8 | Pralatrexate | 0.270 | 0.037 |
| HDAC8 | Lomustine | 0.259 | 0.046 |
| HDAC9 | By-Product of CUDC-305 | -0.493 | 0.000 |
| HDAC9 | Simvastatin | 0.365 | 0.004 |
| HDAC9 | Clofarabine | -0.346 | 0.007 |
| HDAC9 | Pelitrexol | -0.342 | 0.007 |
| HDAC9 | Cytarabine | -0.315 | 0.014 |
| HDAC9 | Palbociclib | -0.295 | 0.022 |
| HDAC9 | Amonafide | -0.291 | 0.024 |
| HDAC9 | O-6-Benzylguanine | 0.286 | 0.027 |
| HDAC9 | Lenvatinib | 0.283 | 0.028 |
| HDAC9 | Cladribine | -0.280 | 0.030 |
| HDAC9 | AT-13387 | -0.279 | 0.031 |
| HDAC9 | 8-Chloro-adenosine | -0.278 | 0.032 |
| HDAC9 | Asparaginase | -0.270 | 0.037 |
| HDAC9 | Fluorouracil | -0.265 | 0.041 |
| HDAC9 | Pemetrexed | -0.265 | 0.041 |
| HDAC9 | Decitabine | -0.264 | 0.042 |
| HDAC9 | Buthionine sulphoximine | 0.263 | 0.042 |
| HDAC9 | Methotrexate | -0.261 | 0.044 |
| HDAC10 | Decitabine | 0.349 | 0.006 |
| HDAC10 | Curcumin | 0.329 | 0.010 |
| HDAC10 | Temsirolimus | 0.315 | 0.014 |
| HDAC10 | Vorinostat | 0.312 | 0.015 |
| HDAC10 | Vemurafenib | 0.311 | 0.015 |
| HDAC10 | Nelarabine | 0.308 | 0.017 |
| HDAC10 | 5-fluoro deoxy uridine 10mer | 0.304 | 0.018 |
| HDAC10 | Chlorambucil | 0.294 | 0.023 |
| HDAC10 | Acrichine | 0.284 | 0.028 |
| HDAC10 | Pipobroman | 0.282 | 0.029 |
| HDAC10 | Cladribine | 0.276 | 0.033 |
| HDAC10 | Thiotepa | 0.263 | 0.042 |
| HDAC10 | Triethylenemelamine | 0.262 | 0.043 |
| HDAC10 | Triapine | 0.259 | 0.046 |
| HDAC10 | Raltitrexed | 0.257 | 0.047 |
| HDAC11 | Carmustine | -0.481 | 0.000 |
| HDAC11 | Oxaliplatin | -0.461 | 0.000 |
| HDAC11 | Ifosfamide | -0.427 | 0.001 |
| HDAC11 | Imexon | -0.418 | 0.001 |
| HDAC11 | Lomustine | -0.418 | 0.001 |
| HDAC11 | BN-2629 | -0.418 | 0.001 |
| HDAC11 | Eribulin mesilate | -0.410 | 0.001 |
| HDAC11 | Nandrolone phenpropionate | -0.402 | 0.001 |
| HDAC11 | Epirubicin | -0.388 | 0.002 |
| HDAC11 | Actinomycin D | -0.387 | 0.002 |
| HDAC11 | XK-469 | -0.384 | 0.002 |
| HDAC11 | Homoharringtonine | -0.368 | 0.004 |
| HDAC11 | LDK-378 | -0.365 | 0.004 |
| HDAC11 | Cyclophosphamide | -0.363 | 0.004 |
| HDAC11 | Palbociclib | -0.358 | 0.005 |
| HDAC11 | Dimethylaminoparthenolide | -0.357 | 0.005 |
| HDAC11 | Carboplatin | -0.355 | 0.005 |
| HDAC11 | Dexrazoxane | -0.353 | 0.006 |
| HDAC11 | kahalide f | 0.352 | 0.006 |
| HDAC11 | Buthionine sulphoximine | -0.349 | 0.006 |
| HDAC11 | Vinblastine | -0.347 | 0.007 |
| HDAC11 | Vinorelbine | -0.347 | 0.007 |
| HDAC11 | Pipobroman | -0.347 | 0.007 |
| HDAC11 | Valrubicin | -0.346 | 0.007 |
| HDAC11 | Arsenic trioxide | -0.346 | 0.007 |
| HDAC11 | AP-26113 | -0.345 | 0.007 |
| HDAC11 | Alvocidib | -0.343 | 0.007 |
| HDAC11 | Paclitaxel | -0.341 | 0.008 |
| HDAC11 | Etoposide | -0.336 | 0.009 |
| HDAC11 | Batracylin | -0.333 | 0.009 |
| HDAC11 | Teniposide | -0.332 | 0.009 |
| HDAC11 | Fenretinide | -0.330 | 0.010 |
| HDAC11 | Rebimastat | -0.330 | 0.010 |
| HDAC11 | Carfilzomib | -0.328 | 0.011 |
| HDAC11 | Bendamustine | -0.325 | 0.011 |
| HDAC11 | Daunorubicin | -0.321 | 0.012 |
| HDAC11 | Idarubicin | -0.319 | 0.013 |
| HDAC11 | 3-Bromopyruvate (acid) | -0.318 | 0.013 |
| HDAC11 | Entinostat | -0.316 | 0.014 |
| HDAC11 | Pipamperone | -0.313 | 0.015 |
| HDAC11 | Hydroxyurea | -0.311 | 0.016 |
| HDAC11 | Dolastatin 10 | -0.311 | 0.016 |
| HDAC11 | Depsipeptide | -0.306 | 0.017 |
| HDAC11 | By-Product of CUDC-305 | -0.302 | 0.019 |
| HDAC11 | Doxorubicin | -0.301 | 0.019 |
| HDAC11 | PX-316 | -0.300 | 0.020 |
| HDAC11 | Imatinib | -0.298 | 0.021 |
| HDAC11 | Mithramycin | -0.296 | 0.021 |
| HDAC11 | Asparaginase | -0.287 | 0.026 |
| HDAC11 | Tamoxifen | -0.287 | 0.026 |
| HDAC11 | Melphalan | -0.285 | 0.027 |
| HDAC11 | Chelerythrine | -0.281 | 0.030 |
| HDAC11 | Nitrogen mustard | -0.272 | 0.036 |
| HDAC11 | BEN | -0.271 | 0.036 |
| HDAC11 | Nilotinib | -0.267 | 0.039 |
| HDAC11 | Parthenolide | -0.264 | 0.041 |
| HDAC11 | Chlorambucil | -0.262 | 0.043 |
| HDAC11 | Irofulven | 0.261 | 0.044 |
| HDAC11 | Thiotepa | -0.261 | 0.044 |
| HDAC11 | Docetaxel | -0.258 | 0.047 |

Cor: Correlation coefficient
